# Personality traits, panel tenure, survey topic, and context as predictors of survey nonresponse patterns in high-frequency online longitudinal surveys

**DOI:** 10.1371/journal.pone.0332902

**Published:** 2025-09-22

**Authors:** Htay-Wah Saw, Arie Kapteyn

**Affiliations:** 1 Michigan Program in Survey and Data Science, Survey Research Center, Institute for Social Research, University of Michigan-Ann Arbor, Ann Arbor, Michigan, United States of America; 2 Center for Economic and Social Research, University of Southern California, United States of America; National Cheng Kung University, TAIWAN

## Abstract

An extensive literature studies the relation between demographic and socio-economic characteristics and attrition in longitudinal studies. In this study, we analyze the independent effects of non-demographic variables—respondent personality traits, panel tenure, and survey topics, using unique datasets from two recently completed high-frequency online longitudinal studies conducted in the U.S. We used latent class analysis to group respondents into various classes based on similarities in their nonresponse patterns across all survey waves, which revealed substantial variations in patterns of nonresponse. Our results indicate that respondent personality traits were strong predictors of nonresponse patterns. Specifically, conscientiousness is positively associated with a lower likelihood of wave nonresponses. In contrast, more open, extroverted, neurotic, and agreeable respondents are more likely to exhibit higher wave nonresponses, but with effect sizes smaller than that of conscientiousness. We found no significant demographic effects on wave nonresponse in one of the studies focused on aging and well-being. However, in the study primarily focused on COVID-19-related topics conducted during the pandemic, we found a few significant demographic effects. Collectively, our findings suggest that personality traits may play a more significant role than conventional demographic and household variables in predicting nonresponse patterns in high frequency (at least one survey per month) online surveys.

## Introduction

Probability-based Internet panels offer researchers the flexibility to launch surveys at any time and collect data from a large number of respondents within a short timeframe, while also offering convenience to respondents who can complete surveys at their own pace and in a private setting. This approach is cost-effective as it eliminates or minimizes the need for intensive fieldwork and logistical operations commonly associated with in-person and telephone interviews, and mail questionnaires. Furthermore, online data enhances statistical efficiency by avoiding the clustering effect often encountered in face-to-face interviews. These advantages have been well-documented in previous studies [1–12].

The Internet technology also allows for quick responses to new and emerging developments. For instance, during the COVID-19 pandemic, online panels have proven valuable in monitoring daily mood changes, observing changes in infection rates, job loss, food insecurity, and vaccination rates over time, and exploring the evolving correlation between COVID-19 vaccines and mental distress in the U.S. adult population [5,13,14]. Similarly, during the H1N1 pandemic, online panels played a crucial role in monitoring population trends related to risk behaviors and vaccine intentions in the U.S. [15]. Additionally, internet panels offer researchers the ability to collect high frequency longitudinal data. This also has a methodological advantage because collecting information closer in time to the occurrence of important events, and collecting it repeatedly reduces the role of measurement error and omitted variables, enabling sharper statistical inference. This is particularly important for minimizing memory errors, a key source of measurement error in survey data [16].

Despite the inherent strengths, as with any longitudinal study, nonresponse and attrition pose a significant challenge in online data collections. Reasons for respondents discontinuing their participation may include, among others, a lack of sustained commitment, panel fatigue or cumulative burden, and external shocks such as illness or loss of employment [17–19].

Both nonresponse and attrition lead to a reduced sample size and decreased precision of survey estimates. Given that data are often collected more frequently in an online panel context, even a modest attrition rate in each wave can lead to a significant loss of sample in a short period of time. Additionally, if the characteristics of panelists who drop out differ from those who continue participating, attrition may introduce bias, which can undermine the validity and generalizability of research findings [18,20–26].

The implications of these challenges are underscored by several studies. Research has shown that statistical adjustments did not completely eliminate bias resulting from respondents not completing all longitudinal surveys [27] and that nonresponse adjustments based on standard demographic variables were not sufficient to fully address attrition bias in panel surveys [28,29]. Additionally, some statistical estimates can be sensitive to the type of adjustment method employed [30–32].

In this study, we analyze survey nonresponse data from two recently completed studies that collected high frequency online longitudinal data. These two studies were implemented in the Understanding America Study (UAS) – a probability-based Internet panel representative of US adults and used for conducting social science, policy, and health-related research. The two studies mainly differ in terms of survey topics (COVID-19 vs. Non COVID-19) and timing of implementation (one during COVID-19 and the other partly during a non COVID-19 period). Additionally, the two studies collected data at various frequencies (every 2 weeks vs. one month) with varying observation periods (14 months vs. 52 months). While there is no universally accepted definition of what qualifies as “high” frequency, we define high-frequency online longitudinal studies as those that field at least one survey per month. We utilize a latent class analysis approach to group respondents based on their similarities in nonresponse patterns. We then estimate multinomial logistic regression models utilizing background information available for all respondents as regressors to predict these nonresponse patterns. The background information we used in the regression analyses include psychological traits, demographics, and health outcomes. We then analyze the independent effects of respondent personality traits and survey topic and context, in addition to conventional demographics. In addition, we analyze the independent effect of respondents’ time in the panel (referred to as panel tenure) in predicting nonresponse patterns due to its importance for panel management and relevance in predicting nonresponse outcomes in online panel surveys [12]. Finally, we compare and contrast the regression coefficients between the two studies and examine whether COVID-19 related topics and the general societal environment during the pandemic moderate the relationship between individual regression coefficients and nonresponse patterns. Findings from the study shed new light on survey nonresponse patterns, along with their correlates and underlying response mechanisms, specific to high-frequency online longitudinal data collections in the US context.

## Background

### Differences between offline and online panel studies

While researchers have been collecting offline panel data for decades, it is only in recent years that the collection of high-frequency online longitudinal data has begun, made possible primarily by the high penetration of the Internet and recent advances in Internet technology. As a consequence, the majority of prior studies analyzing nonresponse and attrition outcomes have focused on offline longitudinal studies [33–35], with limited comparable research using nonresponse data from probability-based online longitudinal studies [12,29,36].

Offline longitudinal studies differ from their online counterparts in many design aspects, and these differences can have different implications for nonresponse and attrition outcomes and the underlying mechanisms influencing them. Table 1 highlights some of the key differences. In the following discussion, we examine these differences in detail.

**Table 1:** Key differences between probability-based online panel vs. offline panel studies.

	Online panel	Offline panel
Primary mode of data collection	Online	• Face-to-face • Mail • Telephone • Sequential mixed modes
Data collection frequency	Very frequent: • Daily • Weekly • Monthly • Bimonthly	Less frequent: • Annually • Biannually • Or longer
Interview length	• 15–30 minutes	• 80–120 minutes
Questionnaires and survey topics across waves	• Mostly varying	• Mostly fixed
Common reason(s) for nonresponse and attrition	• Refusal • Panel fatigue	• Noncontact • Panel fatigue • Refusal

The mode of data collection is the first design aspect that distinguishes online panels from offline panel studies. In most online panel studies, the sole mode of data collection used is the Internet, although some Internet panels offer alternative modes in addition to the online option. For instance, the AmeriSpeak Panel in the U.S. uses a mixed-mode design by providing a telephone option to panelists without Internet access and to panelists with Internet access who are unwilling to provide an email address [37]. Similarly, the GESIS panel in Germany provides paper-and-pencil questionnaires sent via postal mail to participants unable or unwilling to complete surveys online [38]. Providing a second mode option to online panel respondents undermines the cost and efficiency advantage of the online mode but has the potential to improve sample representation.

A few offline panel studies have switched to or begun experimenting with push-to-web sequential mixed modes [39–48]. This shift from a single mode to a mixed-mode design is mainly necessitated by the need to address the rising costs associated with face-to-face and telephone interviews and to take advantage of increasing Internet penetration. In this mixed-mode design, offline panel respondents are encouraged to complete interviews online first, followed by face-to-face or telephone interviews if necessary. This approach substantially reduces costs through decreased fieldwork and fewer field interviews. Couper and McGonagle (2019) [49] provide a comprehensive review of recent developments in the use of web surveys in mixed-mode data collection for conventional offline longitudinal studies.

The two study approaches also differ in terms of data collection frequency. In online panel studies, data collection can be very frequent. Participants might be asked to provide data daily (as in 7-day wellbeing and diary surveys), weekly, monthly, or bimonthly. Participants in Ecological Momentary Assessment (EMA) studies may be interviewed multiple times a day over several days [50,51]. The high frequency is facilitated by the convenience and accessibility of online surveys. In EMA studies, surveys may also be delivered via push notifications through smartphone apps, where timely response is critical and email alone may not suffice. In contrast, offline panel studies generally have less frequent data collection schedules. Data might be collected annually, biannually, or even less frequently. The differences in data collection frequency have significant implications for the type of data gathered and the responsiveness of a study to new developments such as pandemics and financial crises.

Online panel interviews are typically shorter, ranging from 15 to 30 minutes. This brevity is designed to maintain participant engagement and reduce the likelihood of break-off and survey fatigue. In contrast, aided by the presence of interviewers and long between-wave gaps, offline panel interviews tend to be much longer, lasting between 80 and 120 minutes.

Online and offline studies use different questionnaire designs across survey waves. Most online panels field both longitudinal and cross-sectional surveys, with survey topics and questionnaires varying significantly across different waves. This flexibility allows online studies to adapt to changing research needs and emerging topics of interest such as the COVID-19 pandemic. In contrast, offline studies primarily field longitudinal questionnaires with mostly fixed topics across waves, providing consistent data over time. Occasionally, offline panel studies may field supplemental questionnaires between waves to address specific research questions or capture additional data points.

### Different correlates of attrition and response mechanisms in offline vs. online panel studies

The aforementioned design differences can differentially impact nonresponse and attrition outcomes, and the correlates influencing these outcomes may vary between the two study approaches. In any follow-up survey in an offline study, interviewers must first locate respondents and then establish contact with them, conditional on being located. Once contact is made, interviewers attempt to gain cooperation from respondents. Failure at any of these three steps could result in nonresponse. As such, most offline studies have modelled nonresponse and attrition outcomes as a function of whether: sample respondents moved between survey waves, contacts with sample respondents were established conditional on respondents being located, and sample respondents cooperated when approached by interviewers, conditional on contact being made. Non-location occurs when participants move and cannot be reached, while noncontact refers to the difficulty in reaching participants physically despite repeated attempts. These three processes can be modeled separately [35,52–54] or cumulatively without making any distinction among the three processes [28,55].

Given the three aforementioned processes through which nonresponse could occur in offline studies, many prior studies have focused on identifying correlates that predict non-location, non-contact, or the cumulative final outcome [33–35,52–54,56]. Household-, individual-, and interviewer-level variables that have been found to predict these outcomes include, among others, housing arrangement (e.g., renting vs. owning), housing characteristics (e.g., gated entry, single home, communal housing), housing tenure (i.e., years at current residence as proxy for stability), household size, number of children, employment status (e.g., retired, currently working), marital status (as a proxy for geographical stability), changes in events between waves such as changes in employment and household composition [57], level of interviewer experience, interviewer training, and use of the same interviewers across survey waves. However, these household-, individual-, and interviewer-level variables primarily affect attrition through contactability and securing initial cooperation with respondents, making them less relevant for predicting nonresponse patterns in online panel contexts, where surveys are delivered via email and self-administered.

The use of different data collection modes between the two study approaches can impose varying levels of survey burden, which can, in turn, lead to different nonresponse patterns. For instance, since most offline panels use face-to-face and/or telephone modes administered by interviewers, the cognitive effort required to answer difficult or unclear questions may be lower for offline panel respondents as interviewers can clarify questions. Conversely, when answering sensitive questions, the presence of interviewers can create psychological stress for offline panel respondents [58,59].

In this study, we analyze the independent effects of non-demographic variables – respondent personality traits, panel tenure, and survey topics and context – that are likely to be important for predicting nonresponse patterns in high-frequency online longitudinal studies, as detailed below.

### Personality traits as predictors of nonresponse

Online surveys are typically delivered via email and self-administered, which reduces the impact of traditional predictors such as contactability and interviewer interactions. We may hypothesize that participants might decline to continue if the frequency of surveys is considered too high, or due to a lack of interest, or disruption in their survey-taking routine. Based on this expectation, we hypothesize that personality traits are important predictors of a respondent’s likelihood to participate consistently in online panel surveys and may have more explanatory power than conventional demographic and household variables commonly found in offline panel studies.

Building on existing theoretical foundations such as the Social Isolation Hypothesis [60], Saßenroth (2013) [61] emphasizes the predictive power of personality traits over traditional socio-demographic factors, particularly highlighting how subjective experiences influence survey participation decisions. This perspective underscores the role of personality traits as critical determinants of nonresponse patterns in high-frequency online longitudinal studies.

The Big Five personality traits – conscientiousness, openness, agreeableness, neuroticism, and extraversion – are widely used in psychological research and have been found to be stable throughout a person’s lifespan and across cultures and languages [62–65]. In this study, they were measured using a 44-item standardized self-report questionnaire administered to respondents [65–67]. Respondents with certain personality profiles may be more inclined to engage regularly and provide complete data, while others may be prone to drop out or respond sporadically. As such, there is a need to look beyond conventional demographic information in analyzing nonresponse patterns in high-frequency online longitudinal surveys.

This argument is supported by drawing a parallel to the literature on medication adherence, where certain personality traits, particularly conscientiousness, have been shown to play a significant role. The conscientiousness trait is defined as “socially prescribed impulse control that facilitates task- and goal-directed behavior, such as thinking before acting, delaying gratification, following norms and rules, and planning, organizing, and prioritizing tasks” [68]. Individuals high in conscientiousness exhibit qualities such as diligence, perseverance, tenacity, orderliness, and industriousness. These traits are crucial for adhering to medication schedules, as they involve remembering to take medications, planning around dosage times, and maintaining a consistent routine. The effect of conscientiousness in predicting medication adherence has been found to be robust across various disease conditions, population subgroups, and cultural settings [69–75].

Similarly, this characteristic is likely relevant for survey completion in high-frequency online longitudinal studies. Completing online surveys regularly requires respondents to pay attention to survey invitations sent via emails, remember due dates, and respond to survey reminders. Conscientious individuals are likely to excel in these areas due to their propensity for planning, organizing, and prioritizing tasks. Therefore, just as conscientiousness is a strong predictor of medication adherence, it should also serve as a strong predictor of nonresponse patterns characterized by low wave nonresponses in high-frequency online longitudinal studies.

In contrast to conscientiousness, a higher score in openness may reflect individuals’ tendency to embrace new ideas and experiences, but this quality may not translate into a strong commitment to complete high-frequency and longitudinal surveys. More agreeable individuals are trusting, altruistic, sympathetic, and cooperative, which may predict participation in a new one-time study but may not be as strong a predictor of sustained participation in a long-term, continuous study, such as a high-frequency longitudinal survey. Neurotic individuals, being susceptible to anxiety and depression, could be more prone to panel fatigue or study dropout. Extraversion is mainly associated with being talkative, sociable, energetic, and friendly, but high extraversion may not translate to long-term commitment needed for participation in an online longitudinal study. Consequently, we expect openness, agreeableness, neuroticism, and extraversion to be positively associated with higher wave nonresponses in a high-frequency online longitudinal study.

Several studies in the economic and psychological literature have extensively documented personality traits as independent predictors (net of other important factors) and causes of significant life outcomes, including educational attainment, employment outcomes, wealth, mental and physical health status, well-being, marriage, divorce, criminal activity, volunteering, and productivity, among others [76–82]. If personality traits are found to predict nonresponse patterns in high-frequency online longitudinal studies, even after accounting for conventional respondent- and household-level demographic variables, this could have practical implications for how nonresponse adjustment weights are calculated.

In offline panel studies, a few investigations have found that personality traits independently predicted survey completion. Satherley et al. (2015) [83] analyzed the first four waves of response data from the New Zealand Attitudes and Values Study, an offline longitudinal national panel where respondents were interviewed annually using self-administered paper questionnaires, with a web option provided in waves 3 and 4. They found that conscientiousness was positively associated with consistent participation in the panel study. Similarly, Hansson et al. (2018) [26] analyzed response data from the first three waves of the Swedish longitudinal population-based Health, Ageing, and Retirement Transitions in Sweden (HEARTS) study, an offline panel study with annual follow-ups using both paper-pencil questionnaires and online modes. They found that higher extraversion and neuroticism, and lower agreeableness, independently predicted attrition. However, given that online and offline panel studies differ in many design aspects (see Table 1), it is unclear to what extent these findings can be generalized to the context of high-frequency online longitudinal data collection.

Within online panel contexts, Cheng et al. (2020) [29] analyzed wave-to-wave response data from panel surveys fielded in the UAS and found that conscientiousness and openness to experience predicted the incidence of unit nonresponse in subsequent survey waves, even after controlling for cognitive ability and demographic characteristics. Additionally, Lugtig (2014) [18] analyzed response data from surveys conducted in the LISS panel in the Netherlands and found that respondents who dropped out were less likely to be conscientious and more likely to be extraverted and agreeable.

Recent studies in the public opinion literature have begun to explore the role of personality in survey participation. Hibbing et al. (2019) [84] show that traits like openness and conscientiousness are linked to response styles that may bias survey estimates. Valentino et al. (2020) [85] find that openness and extraversion are negatively associated with participation in online surveys compared to face-to-face modes, suggesting potential personality-driven selection biases. While these studies rely on cross-sectional or dual-mode data, our study extends this work by using a richer 44-item personality battery and panel data to examine how personality traits predict actual nonresponse patterns over time in high-frequency online longitudinal surveys.

### Panel tenure and nonresponse patterns

Panel tenure, defined as the length of time a respondent has been part of an online panel, is a crucial factor in understanding and predicting nonresponse patterns in online data collection contexts. Over time, respondents who have been part of a panel for longer periods may develop a stronger sense of commitment and familiarity with the survey process, which can lead to higher response rates and lower attrition rates. Likely, attrition is selective, whereby individuals who are more inclined to participate may be more likely to remain in the panel. By including personality traits—particularly conscientiousness—as predictors in our analysis, we are able to partially account for the heterogeneity in the propensity to respond to surveys, acknowledging that we cannot cleanly separate the roles of selectivity and panel tenure. In what follows, panel tenure therefore is taken to reflect both possible habituation in panel response and the difference in unobserved characteristics between respondents with different tenure lengths.

New panel respondents may not have yet developed the routine of regularly taking surveys in an online environment. Previous studies have shown that prior survey experiences are predictive of cooperation in subsequent surveys, indicating that the early survey experiences of fresh respondents are crucial for their continued participation [28,86]. Positive initial experiences can foster a sense of engagement and commitment, leading to longer panel tenure and reducing the likelihood of nonresponse and attrition.

Most importantly, studies have found that individuals who drop out during the first membership year have distinct demographic, psychological, and psychosocial characteristics compared to those who leave in subsequent years [22,26].

**Fig 1:**
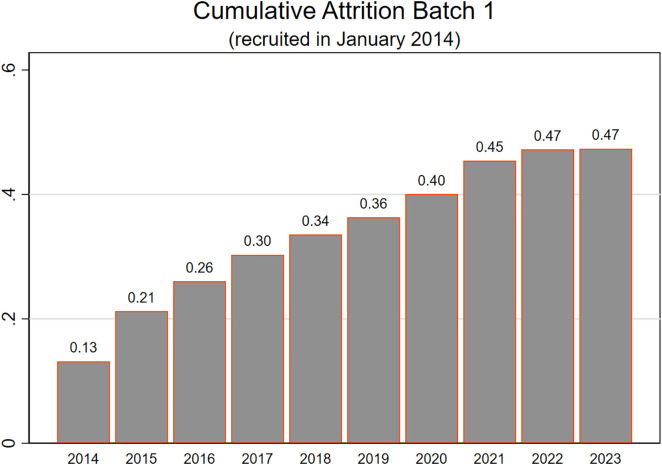
Attrition over time of the first UAS batch recruited in 2014.

Fig 1 illustrates how attrition decreases over time. It shows the cumulative attrition for the first of respondents recruited into the UAS in 2014. One observes that in the first year, 13% of the new respondents dropped out. In the second year, an additional 8% dropped out, and an additional 5% in the third year. By the end of the observation period annual attrition had fallen to just a few percent per year. Batches that were recruited in later years show similar patterns, although attrition levels have gone up over time, in line with general experience in panel studies.

The pattern suggests that the most productive effort in maximizing retention may be early on in a respondent’s tenure. By analyzing panel tenure effects, researchers can identify factors that contribute to quick dropout and apply these insights to newer panel members. Understanding the impact of panel tenure also can help inform the design of nonresponse adjustment weights. In our panel, respondents are invited to participate in a sequence of 22 core surveys, administered biennially, and are also invited to topical surveys such as the COVID-19 series. Although there may be slight variation in the number of ad hoc survey invitations, the overall exposure to survey requests is broadly similar across respondents. For this reason, we consider panel tenure (i.e., time in the panel) to be a reasonable proxy for survey experience.

### Survey topics and context effects

Leverage-salience theory, which incorporates both survey features and respondent characteristics, suggests that different survey features (e.g., survey topics) have varying effects (“leverages”) on participation decisions based on respondents’ socioeconomic characteristics, lifestyles, and preferences [87]. The relevance and interest of survey topics in influencing survey participation decisions, as predicted by leverage-salience theory, have mainly been examined in cross-sectional studies [88,89].

Understanding the role of survey topics and context in online longitudinal studies is important for several reasons. First, longitudinal data collection requires sustained participation over time, and varying survey topics may differentially impact respondents’ willingness to continue participating. Second, as online panels become an increasingly popular method for data collection, identifying factors that influence long-term engagement and reducing nonresponse is essential for maintaining data quality and representativeness. The COVID-19 pandemic has provided a unique opportunity to examine how a highly topical and relevant issue may influence nonresponse patterns.

In this study, we analyze two novel datasets generated from two recently completed online longitudinal studies. These studies primarily differ in their survey topics and contextual factors. Specifically, one study focused on well-being topics both before and during the COVID-19 pandemic, while the other solely addressed COVID-19-related topics and was fielded when COVID-19 was declared a public health emergency in March 2020. The importance and relevance of the COVID-19 study were clearly emphasized to potential participants in the consent survey by including the following text:

*“We are launching a new survey series to help us track how U.S. residents are coping with the coronavirus pandemic. Participants will be asked to fill out a survey every two weeks. We are interested in learning how life changes or stays the same over time for U.S. residents and their family and friends. For that reason, we hope you will join the project so that we have input from every part of the country and from every walk of life. The survey will focus on what is happening in your life, and what you may be doing the same or differently now. We want to learn about the impacts of the pandemic on your family, your job or income, and how you are coping with the changes that have been and continue to be made to help contain the virus.”*

We leverage the timing and relevance of the COVID-19 study, along with the disproportionate effects the pandemic had, to conduct a comparative analysis of survey nonresponse patterns and their correlates between the two studies. On the one hand, we expect the COVID-19 survey to attract a large number of participants, as it was very topical during the pandemic. On the other hand, nonresponse rates in the COVID-19 survey may be higher among subgroups of respondents whose lives and livelihoods were disproportionately affected by the pandemic.

While we did not begin with strong directional hypotheses regarding potential moderating effects of panel topic, the COVID-19 context provided a unique opportunity to explore whether broader societal conditions and topic salience could moderate the relationship between individual characteristics and survey participation. Our analyses of topic effects are primarily exploratory, but the observed differences between the two studies suggest that contextual factors may meaningfully influence nonresponse patterns.

In summary, this study makes several contributions to the emerging literature on nonresponse and attrition in high-frequency online longitudinal data collections. First, it contributes to our understanding of how design differences between offline and online panels differentially influence nonresponse, attrition outcomes, and underlying mechanisms. Second, it offers novel insights into the critical role of respondent personality traits, particularly conscientiousness, in predicting nonresponse patterns in these contexts, even when accounting for other predictors, which could have implications for the construction of nonresponse adjustment weights. Third, by leveraging data from two recent and unique datasets, the study provides new evidence on the independent effect of panel tenure and the potential moderating effect of survey topics on nonresponse patterns.

## Methods

### Data and study population

Data are from two recently completed online longitudinal studies conducted in the Understanding America Study (UAS). Established at the University of Southern California (USC) in 2014, the UAS is a nationally representative, probability-based internet panel of US-households of approximately 15,000 respondents aged 18 and above. Respondents answer surveys on a computer, tablet, or smart phone, wherever they are and whenever they wish to participate. Surveys are conducted in English and Spanish. Panel members are recruited through address-based sampling. Prior access to internet is not a pre-requisite to be in the panel; respondents without prior internet access are provided with a computer tablet and broadband internet subscription. Respondents answer surveys once or twice a month covering various substantive topics. Partly as a result of this, the UAS comprises a vast amount of background information on its respondents, including extensive measures of physical and mental health, income, labor force participation, cognitive functioning, and demographics. For more information about the UAS cohort profile, see Kapteyn et al. (2024) [12].

UAS recruitment procedures follow the Tailored Design Method [90], starting with a postcard, next a short recruiting survey with $5 included and $15 promised for return of the recruiting questionnaire. At the end of the questionnaire, respondents are asked for their interest in participating in a longer run study. If interested, but without Internet, they are offered an Internet-enabled tablet. The process includes several reminders and an offer of additional incentives for consenting respondents to start the first Internet survey, if they have not done so within a given time period. Panel members are paid $20 for a thirty-minute survey. The UAS has instituted a “Sleeper Protocol”, which involves several follow-ups with panel members who have not responded to a survey invitation during a specified number of months. The UAS website (https://uasdata.usc.edu/index.php) provides complete information about UAS methodologies, including response and retention rates for every recruitment batch.

### Understanding coronavirus in America study (UCA)

The first data source is from the Understanding Coronavirus in America (UCA) Study [5]. UCA commenced on March 10, 2020, immediately following the World Health Organization’s declaration of COVID-19 as a global pandemic. All active members of the UAS panel were invited to participate in the UCA. About 7,000 UAS participants signed up for the study. The number of active UAS panel members was approximately 10,000 when the UCA study was launched in early 2020. The number has since grown to 15,000. UCA participants received a survey every two weeks. The bi-weekly surveys elicited information on a broad array of socio-economic, behavioral, and health outcomes associated with the COVID-19 pandemic. A total of 28 biweekly survey waves were conducted, spanning the period March 2020 to July 2021. While the UCA continuously added new participants to the study after the first wave, our analysis specifically focuses on the 5,891 participants recruited in the first wave. This approach ensures that we possess complete response data for the entire duration of the study, from the initial wave to the study’s conclusion across all 28 waves. The UCA website (https://uasdata.usc.edu/page/Covid-19±Home) provides additional information about the study, including download information on available micro data files and documentation.

### The UAS monthly events panel surveys

The second data source is from the UAS monthly events surveys conducted among UAS respondents aged 50 and above. The data collection began in July 2019 and concluded in September 2023, spanning 52 waves. Each individual survey was administered at the beginning of the month, with respondents reflecting on their experiences in the preceding calendar month. These surveys collected information about various life events, including personal and health-related experiences such as health shocks, financial events, as well as personal encounters with sickness or the death of close relatives or friends during the previous month, and solicited self-assessments of their health, life satisfaction, pain levels, hours worked, earnings, employment changes, and medical expenditures during that period. Although the study continuously added new participants after the first wave, our analysis focuses on 3,597 participants recruited in Wave 1. This is to ensure that we have complete response data for the full period of the study across 52 survey waves. More details about the monthly events study including download information on available micro data files and documentation, are presented at the study website (https://uasdata.usc.edu/page/UAS±Monthly±Surveys). All data used in this study were accessed on 15/12/2023.

We acknowledge that this 50 + age restriction overlaps with panel topic, which limits our ability to disentangle topic effects from age-related sample composition. However, to address this concern, we conducted a sensitivity analysis by restricting the COVID-19 sample to respondents aged 50 and above. This allowed for a more balanced age comparison between the two samples. The results—presented in the Supporting Information section in S7 Table —indicate that several previously significant effects, especially those related to personality traits, diminished or disappeared in the age-restricted sample. As such, comparisons between the two studies should be interpreted with caution. But the robustness check provides additional clarity about the role of age composition. Additionally, both studies were implemented within the same probability-based online panel, which enhances comparability and internal consistency across the analyses.

### Analytical approaches

#### Latent class analysis (LCA).

We employ LCA to analyze the response data. Specifically, LCA identifies subgroups of individuals (latent classes) by identifying patterns based on categorical latent variables. In survey research, LCA has been successfully used in various applications, including evaluation of survey questions [91], analysis of survey error [92], investigation of satisficing behaviors [93], and nonresponse analysis in both cross-sectional [94] and longitudinal data collections [18,19,95]. We chose LCA for this study because our goal was to classify respondents into meaningful subgroups based on their longitudinal response behavior—such as consistent responders, early dropouts, or gradual attriters—an approach adopted in prior studies by Lugtig (2014) [18], Hu et al. (2020) [19], and others. Identifying these subgroups allows us to inform targeted retention strategies and explore mechanisms underlying panel attrition. LCA models can also be evaluated for model fit.

For each data set, we estimated a series of latent class models, varying the number of classes from 2 to 8. Lugtig (2014) [18] shows that LCA models perform better than other latent class analysis methods, such as latent class growth analysis (LCGA) and growth mixture models (GMM), in fitting longitudinal response data. The categorical indicators used in the LCA model are equal to 1 for respondents to a survey wave and 0 otherwise. In the monthly events study data, there are 52 response indicators, and in the UCA study data, there are 28 response indicators, each indicating response/nonresponse to individual survey waves. Using these response indicators across panel survey waves, the latent class model identifies and groups respondents into several classes based on their similarity in nonresponse patterns.

For model selection, the conventional likelihood-ratio χ2 test with H_1_ as the unrestricted model is invalid and cannot be used because of sparse cells resulting in a distribution that is not Chi-2 distributed. Instead, we employ other model fit criteria to select the final latent class model as recommended by prior studies [18,96]. These criteria include:

(1) Akaike Information Criterion (AIC) and Bayesian Information Criterion (BIC) [97], where lower values of AIC and BIC indicate better-fitting models.
(2) Vuong-Lo-Mendell-Rubin adjusted Likelihood Ratio Test (LMRT) [98], which assesses whether the latent class model with C classes significantly improves the fit compared to the model with (C-1) classes, with a significant LMRT p-value suggesting a better fit for the model with C classes.
(3) Bootstrapped Likelihood Ratio Test (BLRT) [96], which indicates whether the model with (C-1) classes fits significantly worse than the model with C classes, using bootstrapping to estimate the difference in Log Likelihood values between the models, with a significant BLRT p-value indicating a worse fit for the model with (C-1) classes.
(4) Entropy [99], which measures the accuracy of class separation, with entropy values above 0.8 indicating accurate participant assignment to one class.

Finally, we consider both statistical fit and substantive results in selecting a final model. As a first step, we examine the aforementioned statistical fit measures. We then evaluate the substantive interpretability and usefulness of individual classes, as well as the practicality for designing interventions targeting specific groups of respondents based on the chosen classes. For instance, a model with many classes may fail to identify a critical group in need of targeted intervention or include a class that is too small for meaningful intervention. Moreover, with the addition of more classes, the maximum-likelihood (ML) model becomes increasingly unstable, leading to convergence issues in the estimation and making parsimonious models more attractive options. Additional information about model selection in the LCA framework is provided by Nylund et al. (2007) [96] and https://www.statmodel.com/topic5.shtml.

### Predicted posterior probabilities and latent class membership

After fitting the LCA model with the chosen number of classes, we proceed to generate predicted posterior probabilities for each class for every respondent and assign class membership to the class with the highest predicted probability for each respondent. We present summary statistics of predicted posterior probabilities grouped by predicted class membership to initially assess the accuracy of class classification. Subsequently, we plot observed response probabilities by class membership across survey waves. Alternatively, we can also plot the predicted posterior probabilities. Both approaches however yield similar graphical representations of nonresponse patterns across survey waves.

### Predictor variables

#### Big five personality traits.

The questionnaire for measuring big five personality traits consisted of 44 statements [65–67] related to each of the five traits, with respondents asked to rate their agreement or disagreement on a Likert scale, typically ranging from “strongly disagree” to “strongly agree.” For example, statements such as “I see myself as someone who is organized and detail-oriented” were used to assess conscientiousness, while items like “I see myself as someone who is open to new experiences” were used to measure openness. Some items were reverse-coded to ensure consistency in the direction of the scoring. The responses to these statements were then aggregated to create composite scores for each trait, with higher scores indicating stronger expressions of the corresponding trait. The total score ranges for each of the Big Five personality traits were as follows: agreeableness (0–45), conscientiousness (0–45), extraversion (0–40), neuroticism (0–40), and openness (0–50). The personality data was first collected in the initial substantive survey completed by respondents immediately after they completed the baseline demographic survey that is prerequisite for becoming formal panel members. In our analysis, we used data on personality traits from the latest available UAS wave before the start of the analysis period, as this information is collected every two years from the same respondents.

### Panel tenure variable

Official membership in the UAS begins upon completion of the first demographic survey, also referred to as the My Household survey. To calculate panel tenure, we measured the duration in days between the date the Wave 1 survey was conducted in either of the two longitudinal studies and the date a respondent became an official member of the UAS. For the monthly events and UCA studies, the first waves were fielded on June 3, 2019, and April 1, 2020, respectively. We then grouped respondents into two categories: those with panel tenure less than 1 year (fresh or new panelists) and those with panel tenure of 1 year or more (mature or experienced panelists). This classification enables a comparative analysis of respondent engagement based on their tenure within the panel. The one-year cutoff is meaningful and practical in terms of panel management practices and implementation of targeted interventions.

### Other covariates

Other covariates used in the regression analyses include the following: (1) Hispanic ethnicity (yes, no); (2) Race (white only, black only, others); (3) Gender (male, female); (4) Age groups (18–44, 45–64, 65 and above in the UCA study; 50–64, 65 and above in the monthly event study); (5) Highest educational attainment (up to GED or high school, some college, college and above); (6) Household income (below $50k, $50-$75K, $75K and above); (7) Employment status (currently working, currently not working); (8) Household size (1, 2, 3 and above); (9) Self-reported health (rated on a scale from 1 = excellent to 5 = poor).

Several existing theories justify the use of demographic factors as predictors of survey participation. For example, the Opportunity Cost Hypothesis [100,101] explains that individuals weigh the costs and benefits of survey participation, making socio-demographic factors important in shaping their participation decisions. The Social Exchange Theory [90,102] highlights that the norm of reciprocity and trust in exchanges—often influenced by demographic characteristics such as education or socio-economic status—enhances willingness to participate. Additionally, demographic variables, such as education, can serve as proxies for cognitive burden, where individuals with higher educational attainment or higher socioeconomic status (SES) may find survey tasks less burdensome and are thus more likely to complete them [103–105]. A recent study by Earp et al. (2022) [106] provides additional empirical evidence showing that demographic variables serve as good proxies for respondents’ subjective burden levels that predict survey responses in a national survey.

UAS updates respondents’ demographic and household information every three months. All demographic and household information used in this analysis are from the first wave of data collection in the two respective studies. Data on health outcomes are from the latest available UAS wave as this information is collected every two years on the same respondents.

### Multinomial logistic regression

We estimate MLogit models to predict class membership for each of the two studies. For each multinomial logistic regression estimated, we report average marginal effects (AMEs), 95% CIs, and the level of statistical significance. AMEs represent the average change in the predicted probability of belonging to an attrition class associated with a one-unit change in a predictor variable, holding all other variables constant. All reported p values were adjusted for multiple hypothesis tests using Holm’s method [107]. To account for uncertainty in class membership classification in the latent class models, one could use probabilities predicting class membership for the chosen class as weights in the regression analysis, which effectively assigns higher weights to chosen classes predicted with higher probabilities. We compared both unweighted and weighted regression results and they are similar. This is because all chosen classes in the latent class models were predicted with high probabilities and accuracy, as discussed in the Results section. We present and discuss unweighted regression results in the main text but include weighted results, accounting for uncertainties in classification, in S1 and S2 Tables in the Supporting Information Section for completeness. We then explore how the underlying mechanisms influencing nonresponse patterns, as well as the individual- and household-level variables that predict them, may differ between the two approaches.

Additionally, for each survey wave, the UAS provides survey weights that correct for unequal probabilities of sampling UAS members and align the sample with the U.S. adult population in terms of gender, race/ethnicity, age, education, and geographic location (Census regions) [108]. We decided not to use the UAS survey weights in our main analyses because the focus of this study is to understand attrition dynamics within the panel—specifically, how nonresponse evolves from the original sample over time. This would appear to be most relevant for other similar longitudinal surveys. Using survey weights would make the results most relevant for the unlikely case where one is able to recruit a sample with identical inclusion probabilities, i.e., where the likelihood that an invited panel member agrees to join the panel is identical across individuals. However, for most estimated average marginal effects, we found that the unweighted and weighted regression results are both quantitatively and qualitatively similar. For comparison and completeness, we present the weighted regression results using UAS survey weights in the Supporting Information section (see S8 and S9 Tables).

### Statistical packages

All analyses were conducted using Mplus for Windows Version 8.10. Mplus was mainly utilized for estimating latent class models, generating posterior response probabilities, and classifying class membership [109]. Additionally, Stata for Windows Version 18.0 was used primarily for data management, estimating Multinomial Logistic Regression models for predicting class membership, and generating graphs [110].

### Consent and IRB

Oversight of UAS data collection and dissemination was ceded by University of Southern California IRB (UP-14–00148) to BRANY (Biomedical Research Alliance of New York) IRB (22-030-1044). Participants gave informed consent to participate in the UAS before taking part.

## Results

### Sample summary statistics

Table 2 presents summary statistics for the two samples. We provide mean values for the Big-5 personality traits and self-reported health, and percentages for categorical variables.

**Table 2:** Sample summary statistics by study sample.

	Monthly events study sample (n = 3,597)	COVID-19 sample (n = 5,891)
***Big-5 personality traits***		
Conscientiousness	36.25	35.35
Openness	35.82	35.39
Extroversion	25.64	25.17
Neuroticism	20.52	21.84
Agreeableness	36.27	35.30
***Panel tenure***		
Less than 1 year	13.23%	24.26%
1 year and above	86.77%	75.74%
***Hispanic***		
Yes	6.14%	15.99%
***Race & Ethnicity***		
White only	83.37%	78.83%
Black only	7.47%	8.15%
Others	9.16%	13.02%
**Gender**		
Female	52.32%	57.82%
***Age group***		
18-44	NA	39.02%
45-64	NA	38.02%
50-64	59.55%	NA
65 and above	40.45%	22.95%
***Education***		
GED or High School	23.52%	21.53%
Some College	38.89%	37.11%
College and above	37.59%	41.36%
***HH income***		
Below $50K	42.44%	40.68%
$50-$75K	20.38%	19.84%
$75K and above	37.18%	39.48%
***Employment status***		
Currently working	41.90%	56.13%
Currently not working	58.10%	43.87%
***HH size***		
1	22.85%	17.70%
2	51.32%	39.90%
3+	25.83%	42.40%
***Health status***		
Self-reported health	2.82	2.71

Note: The Big Five personality traits are measured on different scales—agreeableness and conscientiousness range from 0 to 45, extraversion and neuroticism from 0 to 40, and openness from 0 to 50. Mean values should not be compared across traits without accounting for scale differences.

With respect to Big-5 personality traits, there are no discernible differences between the two studies. Relative to the monthly events study, the UCA study attracted a significantly higher percentage of fresh panelists (24.26% vs. 13.23% in the monthly events study) and Hispanics (15.99% vs. 6.14% in the monthly event sample). These differences can be explained by the growth of the UAS and the different time periods covered by the two studies. In the period before March 2020, the sample size of the UAS grew considerably, resulting in more panel members with shorter tenure at the start of the UCA study.

Racial and ethnic compositions are comparable between the two studies. Participation among females was higher in the UCA study compared to the monthly events survey (57.82% vs. 52.32% in the monthly events study). Both studies are comparable in terms of highest educational attainment and household income. The UCA sample had a higher percentage of working individuals (56.13% vs. 41.90% in the monthly events sample), reflecting the age differences between the two studies. We observe differences in household composition between the two samples, with 2-person households accounting for half of the monthly events sample and 3-person households and above making up approximately half of the UCA sample; this difference mainly reflects age differences between the two samples. The mean values for self-reported health are similar between the two samples.

### Latent class model selection

In Table 3, model fit statistics are presented by the number of latent classes separately for the two study samples. Across both samples, the log-likelihood values, AIC, and BIC consistently improve with an increase in the number of classes. However, in the monthly events study sample, the LMRT and BLRT statistics yield conflicting results. The LMRT suggests that a model with 4 classes best fits the data, while the BLRT indicates that a model with 8 classes performs better. Entropy values exceed 0.8 for all latent class models in both samples, indicating high accuracy in class membership assignment. Taken together, these fit statistics suggest that the models with more classes fit the data better. However, considering the substantive interpretability and practicality of implementing targeted interventions, we select latent class models with 5 classes in the monthly events sample and 6 classes in the UCA sample as our final models, highlighted in bold text.

**Table 3:** Model fit statistics by number of response pattern classes.

Monthly events panel study (n = 3,597)									
# of classes	DF^(a)^	LL^(b)^	AIC^(c)^	BIC^(d)^	Entropy^(e)^	LMRT p value^(f)^	BLRT p value^(g)^	Class size (%)	
								Min	Max
2	105	−55856	111923	112573	0.99	0.333	0.000	30%	70%
3	158	−46916	94148	95126	0.99	0.000	0.000	20%	60%
4	211	−43370	87162	88468	0.98	0.000	0.000	12%	55%
**5**✔	**264**	**−41811**	**84150**	**85784**	**0.98**	**0.637**	**0.000**	**11%**	**54%**
6	317	−40684	82002	83963	0.98	0.204	0.000	6%	54%
7	370	−39806	80352	82641	0.97	0.567	0.000	5%	52%
8	423	−39217	79281	81899	0.97	0.206	0.000	5%	51%
COVID-19 panel study (n = 5,891)									
# of classes	DF	LL	AIC	BIC	Entropy	LMRT p value	BLRT p value	Class size (%)	
								Min	Max
2	57	−43924	87962	88343	0.98	0.000	0.000	22%	78%
3	86	−38933	78039	78614	0.96	0.000	0.000	11%	68%
4	115	−37156	74542	75311	0.95	0.000	0.000	6%	64%
5	144	−36573	73434	74396	0.94	0.007	0.000	6%	65%
**6**✔	**173**	**−35988**	**72323**	**73479**	**0.93**	**0.000**	**0.000**	**4%**	**62%**
7	202	−35604	71613	72962	0.93	0.000	0.000	5%	61%
8	231	−35365	71193	72736	0.92	0.008	0.000	3%	61%

(a) DF = Degrees of freedom; ^(b)^ LL = Log-likelihood; ^(c)^ Akaike Information Criterion (AIC)=−2*LL + 2*DF; ^(d)^ Bayesian Information Criterion (BIC)=−2*LL + DF * Ln(N). For LL, larger values indicate better fitting models. In contrast, for both AIC and BIC, lower values suggest better fitting models. ^(e)^ Entropy indicates the extent to which classes can be separated accurately and any entropy values above 0.8 indicates that participants can be accurately assigned to one class; Entropy$1-\frac{\sum_{\text{i}}\sum_{\text{k}}\left(-{\hat{\text{p}}}_{\text{ik}}\text{ln}{\hat{\text{p}}}_{\text{ik}}\right)}{\text{nlnk}},\text{ where }{\hat{\text{p}}}_{\text{ik}}=\text{posterior class probabilities};\text{ k}=\text{number of classes};\text{n}=\text{sample size}$; an entropy value that is close to 1 suggests good classification because the posterior class probabilities are either close to 1 or 0. ^(f)^ LMRT = Lo-Mendell-Rubin adjusted LRT test, which indicates whether the LCA model with C classes is a significant improvement when compared with the previous LCA model with (C-1) classes. ^(g)^ BLRT = Bootstrapped LRT test, similar to LMRT, which indicates whether the LCA model with (C-1) classes fit significantly worse, but now uses bootstrapping to estimate the size of the difference in Log likelihood values between the models with C classes and (C-1) classes. LCA models in bold are selected for predicting class membership based on a combination of their model fit statistics as well as their meaningful substantive interpretations.

Additionally, for sensitivity analysis and completeness, we provide in S1, S2, S3, and S4 Figs in the Supporting Information Section showing observed attrition patterns and in S3, S4, S5, S6 Tables in the Supporting Information Section presenting average marginal effects from multinomial logistic regression results predicting class membership: for the latent class models that are one class smaller and one class larger than the chosen number of latent classes for each of the two study samples, leading to similar substantive conclusions.

### Class membership prediction

Table 4 presents the mean posterior probabilities categorized by predicted class membership. Rows represent class membership, and columns represent the corresponding predicted probabilities. For instance, for the first row in the monthly events data, conditional on being in class 1, the predicted probability of being in classes 1,2,3,4, and 5 are 0.958, 0.000, 0.002, 0.013, and 0.027, respectively. Diagonal values close to 1, and off-diagonal values close to 0, indicate a high level of accuracy in classification. For instance, the mean predicted probabilities for being in classes 1, 2, 3, 4, and 5 are 0.958, 0.988, 0.979, 0.957, and 0.991, respectively, in the monthly events study sample. The mean predicted posterior probabilities across all assigned classes are 0.983 (SD = 0.071, Min = 0.48, Max = 1) for the monthly events study sample and 0.954 (SD = 0.107, Min = 0.352, Max = 1) for the UCA study sample. Overall, the results indicate that classes are predicted with high accuracy, which is consistent with the high entropy values presented in Table 3.

**Table 4:** Classification matrix.

5-class LCA model (Monthly events panel study)						
	Predicted posterior probabilities					
Latent Class	Class 1	Class 2	Class 3	Class 4	Class 5	
1	**0.958**	0.000	0.002	0.013	0.027	
2	0.000	**0.988**	0.012	0.000	0.000	
3	0.002	0.010	**0.979**	0.010	0.000	
4	0.031	0.000	0.011	**0.957**	0.000	
5	0.009	0.000	0.000	0.000	**0.991**	
6-class LCA model (COVID-19 panel study)						
	Predicted posterior probabilities					
Latent Class	Class 1	Class 2	Class 3	Class 4	Class 5	Class 6
1	**0.950**	0.000	0.039	0.006	0.000	0.005
2	0.000	**0.876**	0.019	0.000	0.043	0.062
3	0.047	0.015	**0.886**	0.000	0.000	0.052
4	0.008	0.000	0.000	**0.992**	0.000	0.000
5	0.000	0.007	0.000	0.000	**0.982**	0.011
6	0.002	0.041	0.038	0.000	0.037	**0.881**

### Response patterns in the monthly events study

**Fig 2:**
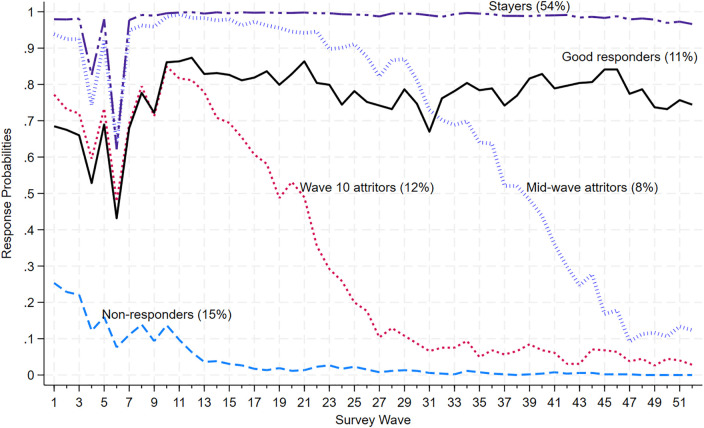
Posterior response probabilities of the 5-class LCA model for the monthly events panel study (n = 3,597).

Fig 2 shows observed posterior response probabilities by class membership across survey waves for the monthly events study. Notably, there were sudden drops in response rates in waves 4 and 6 that were mainly due to a glitch in the case management system, which resulted in failure to send out reminders. This issue was addressed in subsequent waves.

The first group of respondents consists of individuals with response propensities close to 1 across all 52 monthly survey waves. We refer to this group as the “Stayers” group, which comprises approximately 54% of the sample. Participants in this group completed almost all of the 52 monthly survey waves. Similar to the “Stayers” group, the “Good Responders” group, constituting about 11% of the sample, remained until the end of the study but missed a few surveys, with an average response rate of approximately 0.8 across all survey waves.

The “Mid-wave Attritors” group, constituting 8% of the sample, exhibits a response pattern similar to the “Stayers” group until Wave 15, after which nonresponse gradually increased in the Mid-waves, and they did not return. Similar to the “Mid-wave Attritors” group are the “Wave-10 Attritors” group, who make up a significant 12% of the sample and began to leave earlier than the “Mid-wave Attritors” in Wave 10, dropping linearly from an average response rate of about 85% in Wave 8 to about 5% in Wave 31 and then taper off until the end of the study period.

At the lower end of the nonresponse spectrum are a group of respondents with a response probability close to zero right from the beginning. We refer to this group as the “Non-responders” group, making up about 15% of the sample.

In S1 and S2 Figs in the Supporting Information Section, we present nonresponse patterns for the two latent class models: one with a reduced class (i.e., 4 classes) and one with an additional class (i.e., 6 classes) compared to the selected class for comparison and completeness. These nonresponse patterns demonstrate similarities with the patterns in the chosen class, particularly among the “Stayers” and “Non-responders” groups.

### Response patterns in the UCA study

**Fig 3:**
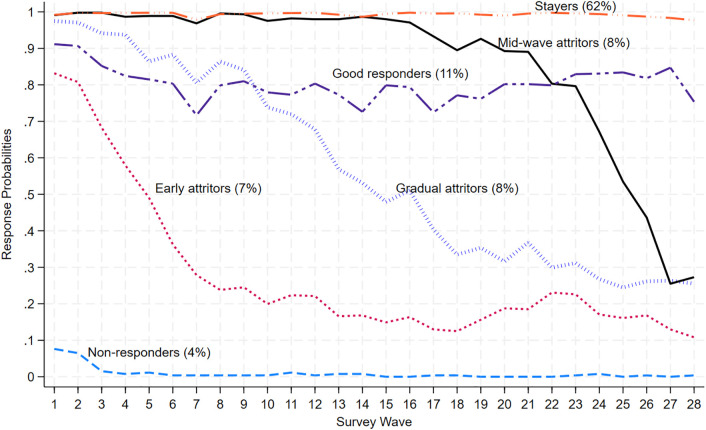
Posterior response probabilities of the 6-class LCA model for the COVID-19 panel study (n = 5,891).

Fig 3 presents observed posterior response probabilities across survey waves by class membership for the UCA study.

There are both similarities and differences in nonresponse patterns and their sample compositions between the two studies. For instance, both studies have a “Stayers” group but their share in the UCA sample is larger than they are in the monthly events sample (62% vs. 54% in the monthly events study). The “Mid-wave Attritors” group in the UCA sample differs from the “Mid-wave Attritors” group in the monthly events sample. Mid-wave leaving respondents in the UCA study responded to all surveys until mid-wave (i.e., Wave 14) and then began to drop out slowly until wave 23, reaching a response rate of about 0.8, with a steep decline afterward until the end of the study period. Their sample share, however, is the same in both samples, accounting for 8% of respondents. The response pattern for “Good Responders” and their sample share (11%) is similar to a large extent in both studies.

The “Gradual Attritors” group, making up 8% of the UCA sample, completed the first survey with a response rate close to 1 and then began to leave gradually and linearly after Wave 1 until the end of the study, eventually dropping to a response rate of 25%. Notably, the response patterns for the “Gradual Attritors” group in the UCA study is similar to that for the “Mid-wave Attritors” group in the monthly events study but they differ in the timing where the gradual attrition begins (Wave 2 vs. Wave 23 in the monthly events study).

The “Early Attritors” group, making up 7% of the UCA study respondents, completed the first two surveys with a response rate of 0.8. Then, their response rate began to decline steeply until Wave 8, where it reached 0.2, tapering off afterward until the end of the study period. Their response pattern is similar to the Wave-10 attrition pattern found in the monthly events study but differs mainly in the timing of the steep decline (Wave 2 vs. Wave 8 in the monthly events study).

Looking at the lowest end of the nonresponse spectrum, the “Non-responders” group with a response rate close to 0 across all waves make up a smaller share of the sample in the UCA study compared to the monthly events study (4% vs. 15% in the monthly events study).

For comparison, in S3 and S4 Figs in the Supporting Information Section, we present response patterns for the latent class model with a reduced class (i.e., 5 classes) and the latent class model with an additional class (i.e., 7 classes), compared to the selected model. These patterns demonstrate similarities with the chosen latent class model.

### Multinomial logistic regression results

Tables 5 and 6 present average marginal effects (AMEs) from multinomial regression models predicting membership classes characterized by various nonresponse patterns in the monthly events and UCA datasets, respectively. As previously mentioned, AMEs represent the average change in the predicted probability of belonging to an attrition class associated with a one-unit change in a given predictor, holding all other variables constant. For example, if the AME for a given predictor is 0.012 for a particular attrition class, this means that a one-unit increase in that predictor is associated with a 1.2 percentage point increase in the probability of being classified into that attrition class, holding all other variables constant. Below, we highlight key differences in the estimated coefficients between the two studies. Significance levels have been adjusted for multiple hypotheses testing for each latent class using Holm’s method. By considering every estimated coefficient as representing a separate hypothesis we are conservative. However, as we are not just interested in the overall effect of for example personality or demographics, but rather in their individual effects, this seems appropriate. The 95% confidence intervals have not been adjusted. The correction for multiple hypotheses testing renders very few of the estimated coefficients significantly different from one in Table 5 except for personality traits (the monthly events survey). In Table 6 (the UCA study), more coefficients (i.e., AMEs), including some demographic variables, differ significantly from zero. In the Discussion and Conclusion section, we provide additional contexts and detailed explanations about these differences.

**Table 5:** Multinomial logistic regressions predicting class membership in the *monthly events panel study*. Reported coefficients are average marginal effects (AMEs), representing the average change in the predicted probability of each outcome category associated with a one-unit change in a given predictor, holding all other variables constant.

	Non-responders	Wave 10 attritors	Mid-wave attritors	Good responders	Stayers
***Big-5 Personality Traits***					
Conscientiousness Score	−0.003 [−0.005,-0.000]	−0.001 [−0.003,0.001]	−0.002 [−0.004,-0.000]	−0.001 [−0.004,0.001]	0.007*** [0.004,0.011]
Openness Score	0.001 [−0.000,0.003]	0.001 [−0.000,0.003]	−0.000 [−0.002,0.001]	0.001 [−0.001,0.003]	−0.003 [−0.006,-0.001]
Extroversion Score	0.004*** [0.002,0.006]	0.000 [−0.002,0.002]	0.000 [−0.001,0.002]	0.000 [−0.002,0.002]	−0.005** [−0.007,-0.002]
Neuroticism Score	0.002 [0.000,0.004]	0.003 [0.001,0.005]	−0.001 [−0.003,0.001]	0.001 [−0.001,0.003]	−0.005** [−0.008,-0.002]
Agreeableness Score	0.001 [−0.001,0.003]	0.003 [0.001,0.005]	0.000 [−0.001,0.002]	0.002 [−0.000,0.004]	−0.006*** [−0.010,-0.003]
***Panel Tenure (Ref: Less than 1 year)***					
1 year and above	−0.034 [−0.071,0.002]	0.005 [−0.028,0.039]	−0.021 [−0.052,0.011]	−0.008 [−0.042,0.026]	0.058 [0.006,0.110]
***Hispanic (Ref: No)***					
Yes	0.058 [0.005,0.112]	−0.002 [−0.048,0.044]	−0.046** [−0.075,-0.017]	0.007 [−0.038,0.052]	−0.017 [−0.087,0.053]
***Race & Ethnicity (Ref: White only)***					
Black only	0.004 [−0.040,0.047]	0.005 [−0.038,0.048]	0.012 [−0.025,0.050]	0.029 [−0.015,0.074]	−0.051 [−0.116,0.014]
Others	0.014 [−0.025,0.053]	−0.012 [−0.048,0.024]	−0.014 [−0.044,0.016]	0.035 [−0.005,0.076]	−0.023 [−0.081,0.034]
***Gender (Ref: Female)***					
Male	0.019 [−0.004,0.042]	0.015 [−0.008,0.038]	0.002 [−0.018,0.021]	−0.013 [−0.035,0.010]	−0.023 [−0.058,0.011]
***Age Group (Ref: 50–64)***					
65 and above	0.016 [−0.011,0.042]	−0.011 [−0.036,0.015]	0.022 [−0.001,0.044]	−0.029 [−0.053,-0.004]	0.002 [−0.038,0.041]
***Education (Ref: GED or high school)***					
Some College	−0.003 [−0.034,0.027]	−0.007 [−0.035,0.022]	−0.001 [−0.025,0.023]	−0.007 [−0.036,0.022]	0.018 [−0.025,0.062]
College and above	−0.031 [−0.063,0.002]	−0.010 [−0.042,0.022]	−0.005 [−0.032,0.023]	−0.017 [−0.049,0.015]	0.063 [0.014,0.111]
***HH Income (Ref: Below $50K)***					
$50-$75K	−0.000 [−0.031,0.030]	0.004 [−0.027,0.036]	0.007 [−0.020,0.033]	−0.027 [−0.056,0.002]	0.017 [−0.030,0.063]
$75K and above	−0.001 [−0.030,0.029]	−0.016 [−0.045,0.013]	−0.009 [−0.033,0.016]	−0.003 [−0.033,0.026]	0.029 [−0.016,0.073]
***Employment Status (Ref: Currently working)***					
Currently not working	0.000 [−0.026,0.026]	−0.001 [−0.027,0.025]	−0.005 [−0.028,0.018]	−0.035 [−0.061,-0.010]	0.041 [0.002,0.080]
***Household Size (Ref: 1)***					
2	−0.019 [−0.048,0.010]	0.001 [−0.027,0.028]	−0.009 [−0.033,0.015]	0.009 [−0.019,0.037]	0.018 [−0.025,0.062]
3 and above	−0.002 [−0.036,0.032]	0.021 [−0.012,0.054]	0.009 [−0.020,0.038]	−0.001 [−0.032,0.030]	−0.027 [−0.077,0.023]
***Health Status***					
	−0.008 [−0.021,0.004]	0.009 [−0.003,0.021]	0.019*** [0.009,0.030]	0.007 [−0.005,0.020]	−0.027* [−0.046,-0.008]
n	3446				

95% confidence intervals in brackets; * p < 0.10, ** p < 0.05, *** p < 0.01. All p values were adjusted for multiple hypothesis tests using Holm’s method [107]. Note the 95% CIs were not adjusted for multiple hypothesis tests.

**Table 6:** Multinomial logistic regressions predicting class membership in the COVID-19 panel study. Reported coefficients are average marginal effects (AMEs), representing the average change in the predicted probability of each outcome category associated with a one-unit change in a given predictor, holding all other variables constant.

	Non-responders	Early attritors	Gradual attritors	Mid-wave attritors	Good responders	Stayers
***Big-5 Personality Traits***						
Conscientiousness Score	−0.000 [-0.001,0.001]	−0.001 [-0.003,-0.000]	−0.003*** [-0.004,-0.001]	−0.000 [-0.002,0.001]	−0.003*** [-0.005,-0.001]	0.008*** [0.005,0.010]
Openness Score	0.001 [-0.000,0.002]	0.002*** [0.001,0.003]	0.001 [-0.000,0.002]	0.001 [0.000,0.003]	−0.000 [-0.002,0.001]	−0.005*** [-0.007,-0.003]
Extroversion Score	−0.000 [-0.001,0.001]	0.002** [0.001,0.003]	0.001 [-0.001,0.002]	0.000 [-0.001,0.001]	0.001 [-0.001,0.002]	−0.003* [-0.005,-0.001]
Neuroticism Score	0.000 [-0.001,0.001]	0.001 [0.000,0.003]	0.001 [-0.001,0.002]	0.001 [-0.001,0.002]	0.001 [-0.000,0.003]	−0.004*** [-0.006,-0.002]
Agreeableness Score	−0.000 [-0.001,0.001]	0.001 [-0.000,0.003]	−0.000 [-0.001,0.001]	−0.001 [-0.002,0.001]	0.003** [0.001,0.004]	−0.003 [-0.005,-0.000]
***Panel Tenure*** ***(Ref: Less than 1 year)***						
1 year and above	0.044*** [0.035,0.053]	−0.011 [-0.026,0.005]	−0.003 [-0.019,0.014]	0.021 [0.006,0.037]	−0.033** [-0.053,-0.013]	−0.019 [-0.048,0.010]
***Hispanic*** ***(Ref: No)***						
Yes	0.088*** [0.060,0.115]	0.003 [-0.014,0.021]	−0.001 [-0.020,0.017]	−0.016 [-0.034,0.003]	0.037** [0.013,0.061]	−0.111*** [-0.149,-0.074]
***Race & Ethnicity*** ***(Ref: White only)***						
Black only	0.019 [-0.006,0.044]	0.030 [0.002,0.057]	−0.016 [-0.039,0.008]	−0.013 [-0.037,0.011]	0.034 [0.000,0.067]	−0.054 [-0.099,-0.008]
Others	0.015 [-0.002,0.032]	−0.003 [-0.021,0.015]	−0.012 [-0.031,0.007]	−0.013 [-0.033,0.007]	0.010 [-0.013,0.033]	0.003 [-0.033,0.039]
***Gender*** ***(Ref: Female)***						
Male	−0.014 [-0.025,-0.004]	0.012 [-0.002,0.027]	0.001 [-0.014,0.016]	−0.004 [-0.019,0.010]	0.006 [-0.011,0.023]	−0.000 [-0.026,0.026]
***Age Group*** ***(Ref: 18–44)***						
45-64	−0.008 [-0.020,0.004]	−0.051*** [-0.068,-0.035]	−0.059*** [-0.078,-0.041]	−0.024 [-0.043,-0.006]	−0.034** [-0.054,-0.014]	0.177*** [0.146,0.208]
65+	0.005 [-0.015,0.024]	−0.061*** [-0.081,-0.040]	−0.079*** [-0.100,-0.058]	−0.043*** [-0.065,-0.021]	−0.044** [-0.071,-0.018]	0.222*** [0.181,0.263]
***Education*** ***(Ref: GED or high school)***						
Some College	−0.011 [-0.026,0.004]	0.008 [-0.010,0.027]	−0.028 [-0.049,-0.007]	0.002 [-0.018,0.022]	0.006 [-0.016,0.027]	0.024 [-0.010,0.057]
College and above	−0.009 [-0.025,0.008]	−0.009 [-0.028,0.010]	−0.034** [-0.056,-0.011]	−0.019 [-0.039,0.002]	−0.011 [-0.034,0.012]	0.082*** [0.045,0.118]
***HH Income*** ***(Ref: Below $50K)***						
$50-$75K	−0.003 [-0.017,0.010]	−0.007 [-0.027,0.012]	−0.014 [-0.033,0.006]	0.006 [-0.014,0.026]	0.011 [-0.013,0.034]	0.008 [-0.027,0.042]
$75K and above	0.009 [-0.006,0.023]	−0.019 [-0.036,-0.002]	−0.009 [-0.028,0.010]	−0.001 [-0.020,0.017]	−0.007 [-0.028,0.014]	0.028 [-0.005,0.060]
***Employment Status*** ***(Ref: Currently working)***						
Currently not working	0.010 [-0.003,0.022]	−0.020 [-0.035,-0.005]	−0.012 [-0.029,0.004]	0.003 [-0.014,0.019]	−0.016 [-0.035,0.002]	0.036 [0.007,0.065]
***Household Size*** ***(Ref: 1)***						
2	−0.009 [-0.025,0.007]	−0.004 [-0.023,0.015]	0.008 [-0.012,0.028]	−0.000 [-0.021,0.020]	0.021 [-0.002,0.043]	−0.015 [-0.050,0.020]
3 and above	−0.002 [-0.019,0.015]	0.008 [-0.012,0.027]	0.022 [0.001,0.042]	0.005 [-0.016,0.026]	0.027 [0.004,0.049]	−0.059** [-0.095,-0.022]
***Health Status***						
Self-report of health	−0.004 [-0.010,0.002]	−0.004 [-0.012,0.003]	−0.001 [-0.009,0.007]	0.005 [-0.003,0.013]	0.001 [-0.008,0.010]	0.003 [-0.011,0.017]
n	5743					

95% confidence intervals in brackets; * p < 0.10, ** p < 0.05, *** p < 0.01. All p values were adjusted for multiple hypothesis tests using Holm’s method [107]. Note the 95% CIs were not adjusted for multiple hypothesis tests.

### Differences by respondent personality traits

Our results in both Tables 5 and 6 show consistent effects of conscientiousness in predicting nonresponse patterns. In the monthly events study, the regression results in Table 5 reveal that respondents with higher conscientiousness scores are less likely to be in the ”Non-responders” and “Mid-wave Attritors” classes and are more likely to be in the “Stayers” class. For instance, our results reveal that a one-point increase in Conscientiousness is associated with a 0.7 percentage point increase in the predicted probability of being classified as a Stayer in the monthly events study, holding all other variables constant. This difference is statistically significant even after adjusting for multiple hypotheses testing. Although the estimated average marginal effects of conscientiousness are negative in the regression models predicting membership in the “Non-respondents”, “Wave 10 Attritors”, “Mid-wave attritors” and “Good Responders” attrition classes in the monthly events study, they are not statistically significant after applying adjustments for multiple hypotheses tests.

Similarly, in the UCA study, results from Table 6 show that respondents with higher conscientiousness scores are more likely to be associated with the “Stayers” class: A one-point increase in conscientiousness score is associated with a 0.8 percentage point increase in the probability of being classified as a Stayer, holding all other variables constant. In contrast, a one-point increase in conscientiousness is associated with a 0.3 percentage point decrease in the predicted probability of being classified as either a “Gradual Attritor” or a “Good Responder”. These differences remain significant even after adjustments for multiple hypothesis tests. For the remaining attrition classes in the UCA study, the estimated average marginal effects for the Conscientiousness trait are negative but are not statistically significant.

It is worth mentioning that without adjustments for multiple hypotheses tests, as done in prior studies such as Lugtig (2014) [18] and Cheng et al. (2020) [29], all estimated average marginal effects for conscientiousness across the attrition classes would have reached statistical significance levels in both study samples. Despite the more stringent and conservative approach we used in handling the estimated regression coefficients, our results still emphasize the importance, robustness, and significance of conscientiousness in predicting nonresponse patterns in high-frequency online longitudinal studies, while minimizing the risk of false conclusions.

In contrast to conscientiousness, individuals with high openness scores are more likely to be associated with the attrition classes than with the “Stayers” class in both studies. This difference is more pronounced in the UCA sample than in the monthly events data. For instance, a one-point increase in the Openness score is associated with a 0.5 percentage point decrease in the probability of being classified as a “Stayer” and a 0.2 percentage point increase in the probability of being classified as an “Early Attritor” in the UCA study (p-value<0.01). Without adjustments for multiple hypothesis tests, the estimated average marginal effects for openness would have been statistically significant across all the attrition classes. However, as previously mentioned, the adjusted results provide a more robust conclusion.

Similar to openness, more extroverted, neurotic, and agreeable respondents are less likely to be associated with the “Stayer” class and more likely to fall into one of the attrition classes. For instance, in the monthly events study, a one-point increase in Extroversion, Neuroticism, and Agreeableness scores is associated with a 0.5 to 0.6 percentage point decrease in the predicted probability of being classified as a “Stayer” (p < 0.05). Comparable effect sizes for these traits were also observed in the UCA study.

Taken together, our results underscore the robustness, importance, and significance of respondent personality traits, particularly conscientiousness, in predicting nonresponse patterns in high-frequency online longitudinal studies. The relationships we found are consistent with our previously stated hypotheses, as well as with the general findings in the medication adherence literature.

### Differences by panel tenure status

The results in Tables 5 and 6 show striking differences in the panel tenure effect between the two studies. The monthly events attrition regression results in Table 5 show no significant differences in average marginal effects by panel tenure across all membership classes. In contrast, the results in Table 6 reveals that, compared to fresh respondents, experienced panelists are approximately 4.4 percentage point more likely to be classified as “Non-responders” and 3.3 percentage points less likely to be classified as “Good responders” (p-value<0.05). No significant panel tenure effects were found for the remaining attrition classes in the UCA study, indicating that experienced respondents who decided to leave the UCA study did so from the beginning.

### Differences among demographic subgroups

**Hispanics**. The regression results in Tables 5 indicate no significant difference between Hispanic and non-Hispanic individuals across all attrition groups in the monthly events study, except that Hispanic respondents are less likely to be classified as “Mid-wave Attritors.” However, the results from Table 6 reveal a different pattern: compared to non-Hispanic individuals, Hispanic respondents are 8.8 percentage points more likely to be classified as “Non-Responders” (p-value<0.01), 3.7 percentage points more likely to be classified as “Good Responders” (p-value<0.05), and 11.1 percentage points less likely to be classified as “Stayers” (p-value<0.01).

**Race**. In both studies, we did not find significant differences in average marginal effects by respondent race across all membership classes.

**Gender**. No significance differences in average marginal effects by respondent gender are found in either of the two studies.

**Age Groups.** In the monthly events study, age has no significant impact. Note however that the age range is restricted to 50 + , a plausible explanation for the absence of age effects. In the UCA study, we observe a large, significant, and negative association between age and nonresponse patterns, with younger participants displaying significantly higher dropout rates compared to their older counterparts across all attrition classes and these differences widen with age. For instance, compared to the 18–44 age group, individuals aged 45–64 and 65 and older are 17.7 and 22.2 percentage points more likely, respectively, to be classified as “Stayers” in the UCA study (p-value<0.01). However, in the sensitivity analysis where we restricted the UCA sample to respondents aged 50 and above, the large and significant age effects disappeared. Detailed results from the age-restricted supplementary analysis are included in the Supporting Information section (S7 Table).

**Education.** In the monthly events study, we find no association between education and membership of any of the attrition classes. In contrast, in the UCA study, individuals with at least a college education are 3.4 percentage points less likely to be classified as Gradual Attritors (p-value<0.05) and 8.2 percentage points more likely to be classified as Stayers, compared to those with at most a GED or high school diploma (p-value<0.01).

**Household Income, Household Size, and Employment Status.** Household income has no effect on nonresponse patterns in either study after controlling for other factors. Household size seems to better predict nonresponse patterns in the UCA study than in the monthly events study. Notably, in the UCA study, respondents from households with at least three people (relative to those in single-person households) are approximately 6 percentage points less likely to be classified as “Stayers.”

### Differences by health status

In the monthly events study, worsening health is associated with an increased likelihood of dropout and is a significant predictor of membership in the “Mid-wave Attritors” group (p-value<0.01), and to a lesser extent, in the “Stayers” group (p-value<0.1) (Note that the rating scale for the self-reported health variable ranges from 1 = Excellent to 5 = Poor). Our analysis of the interaction effects between age and health status show that the health effects are largest among older respondents aged 65 and above (results not shown). This finding aligns with existing literature, which suggests that individuals, particularly in the older age group, with poor health often prioritize healthcare needs over survey participation, leading to higher dropout rates [53,54,111,112].

However, the UCA study presents a contrasting picture, as no discernible association between health status and any of the attrition classes is observed. Additionally, we did not find any interaction effects between age and health status in the UCA study (results not shown). 

It is worth reiterating that the unweighted regression results (i.e., AMEs) presented in Tables 5 and 6 of the main text are both quantitatively and qualitatively similar to their weighted counterparts based on UAS survey weights, as shown in S8 and S9 Tables in the Supporting Information section.

## Discussion and conclusion

In this study, we examined nonresponse patterns and their correlates in two recently completed high-frequency online longitudinal studies that differed primarily in terms of survey topics, frequency, relevance, and target age groups (50+ or 18+). Our findings provide suggestive evidence that design differences between offline and online panels may differentially influence nonresponse and attrition outcomes. Specifically, we found that respondent personality traits play a significant role, while conventional demographic and household variables, which are commonly used in offline panel studies, were often insignificant. Leveraging unique datasets from two studies that primarily differ in topic relevance and interest, we assessed the COVID-19-related context as a potential factor moderating the relationship between individual regression coefficients and nonresponse patterns. Below, we discuss the significance and implications of our findings, provide suggestions for future research directions, and highlight the strengths and weaknesses of our study approach.

### Practical implications related to personality traits

Our findings have several important implications for the design, implementation, and management of online panel surveys. For instance, survey organizations could implement targeted interventions to keep respondents with lower conscientiousness engaged, such as offering frequent reminders via text messages or additional monetary incentives. Additionally, personality traits could be utilized as predictive tools to identify participants at higher risk of attrition, allowing for early intervention before dropout occurs. Providing additional support to individuals high in neuroticism or agreeableness, who may be more prone to attrit, can also enhance their commitment to the study.

In terms of nonresponse adjustment strategies, online survey researchers could consider incorporating personality measures into weighting schemes to improve the accuracy of bias adjustments, given that personality traits like conscientiousness are not only strong predictors of survey participation but also causally linked to significant life outcomes. While population-level benchmarks for personality traits do not exist—making traditional post-stratification infeasible—researchers can still leverage internal benchmarks by collecting personality data from all respondents at baseline. Ideally, this should occur immediately after respondents become panel members or immediately after respondents complete the baseline mandatory demographic survey that is prerequisite for becoming panel members. Having personality data for the full initial sample enables researchers to incorporate these variables into longitudinal weights and track potential attrition-related bias over time.

### Strength and limitations

A key strength of this study is the use of data from two recently completed studies, both conducted within the same probability-based online panel dedicated to social science and health-related research. The inclusion of rich background information for all respondents enhances the precision of statistical inferences, and the availability of Big Five personality traits data allowed us to emphasize the significance of personality factors, particularly in relation to attrition. However, a limitation is that the study does not assess the extent to which selective attrition may result in biases in some key substantive variables. Another limitation is that our results may have limited generalizability beyond the context we examined. Despite this, our findings provide valuable insights into the complexities and unique patterns of nonresponse and attrition, as well as their correlates and underlying mechanisms, in the context of high-frequency online longitudinal data collection—a topic that has received limited attention thus far.

## Supporting information

S1 Fig Posterior response probabilities of the latent class model that is *one class smaller* than the chosen number of latent classes for the *monthly events panel study.* (TIF)

S2 Fig Posterior response probabilities of the latent class model that is *one class larger* than the chosen number of latent classes for the *monthly events panel study.* (TIF)

S3 Fig Posterior response probabilities of the latent class model that is *one class smaller* than the chosen number of latent classes for the *COVID-19 panel study.* (TIF)

S4 Fig Posterior response probabilities of the latent class model that is *one class larger* than the chosen number of latent classes for the *COVID-19 panel study.* (TIF)

S1 Table Weighted multinomial logistic regressions predicting class membership in the *monthly events panel study* using *probabilities predicting class membership for the chosen classes as weights.* Reported coefficients are average marginal effects (AMEs), representing the average change in the predicted probability of each outcome category associated with a one-unit change in a given predictor variable, holding all other variables constant. 95% confidence intervals in brackets; * p < 0.10, ** p < 0.05, *** p < 0.01. All p values were adjusted for multiple hypothesis tests using Holm’s method [107]. Note the 95% CIs were not adjusted for multiple hypothesis tests.
(DOCX)

S2 Table Weighted multinomial logistic regressions predicting class membership in the *COVID-19 panel study* using *probabilities predicting class membership for the chosen classes as weights.* Reported coefficients are average marginal effects (AMEs), representing the average change in the predicted probability of each outcome category associated with a one-unit change in a given predictor variable, holding all other variables constant. 95% confidence intervals in brackets; * p < 0.10, ** p < 0.05, *** p < 0.01. All p values were adjusted for multiple hypothesis tests using Holm’s method [107]. Note the 95% CIs were not adjusted for multiple hypothesis tests.
(DOCX)

S3 Table Multinomial logistic regressions predicting class membership in the m*onthly events panel study* for the latent class model that is *one class smaller* than the chosen number of latent classes. Reported coefficients are average marginal effects (AMEs), representing the average change in the predicted probability of each outcome category associated with a one-unit change in a given predictor variable, holding all other variables constant. 95% confidence intervals in brackets; * p < 0.10, ** p < 0.05, *** p < 0.01. All p values were adjusted for multiple hypothesis tests using Holm’s method [107]. Note the 95% CIs were not adjusted for multiple hypothesis tests.
(DOCX)

S4 Table Multinomial logistic regressions predicting class membership in the *monthly events panel study* for the latent class model that is *one class larger* than the chosen number of latent classes. Reported coefficients are average marginal effects (AMEs), representing the average change in the predicted probability of each outcome category associated with a one-unit change in a given predictor variable, holding all other variables constant. 95% confidence intervals in brackets; * p < 0.10, ** p < 0.05, *** p < 0.01. All p values were adjusted for multiple hypothesis tests using Holm’s method [107]. Note the 95% CIs were not adjusted for multiple hypothesis tests.
(DOCX)

S5 Table Multinomial logistic regressions predicting class membership in the *COVID-19 panel study* for the latent class model that is *one class smaller* than the chosen number of latent classes. Reported coefficients are average marginal effects (AMEs), representing the average change in the predicted probability of each outcome category associated with a one-unit change in a given predictor variable, holding all other variables constant. 95% confidence intervals in brackets; * p < 0.10, ** p < 0.05, *** p < 0.01. All p values were adjusted for multiple hypothesis tests using Holm’s method [107]. Note the 95% CIs were not adjusted for multiple hypothesis tests.
(DOCX)

S6 Table Multinomial logistic regressions predicting class membership in the *COVID-19 panel study* for the latent class model that is *one class larger* than the chosen number of latent classes. Reported coefficients are average marginal effects (AMEs), representing the average change in the predicted probability of each outcome category associated with a one-unit change in a given predictor variable, holding all other variables constant. 95% confidence intervals in brackets; * p < 0.10, ** p < 0.05, *** p < 0.01. All p values were adjusted for multiple hypothesis tests using Holm’s method [107]. Note the 95% CIs were not adjusted for multiple hypothesis tests.
(DOCX)

S7 Table Multinomial logistic regressions predicting class membership in the *COVID-19 panel study*, restricted to respondents *aged 50 and above.* Reported coefficients are average marginal effects (AMEs), representing the average change in the predicted probability of each outcome category associated with a one-unit change in a given predictor variable, holding all other variables constant. 95% confidence intervals in brackets; * p < 0.10, ** p < 0.05, *** p < 0.01. All p values were adjusted for multiple hypothesis tests using Holm’s method [107]. Note the 95% CIs were not adjusted for multiple hypothesis tests.
(DOCX)

S8 Table Weighted multinomial logistic regression results predicting class membership in the *monthly events panel study* using UAS *survey weights.* Reported coefficients are average marginal effects (AMEs), representing the average change in the predicted probability of each outcome category associated with a one-unit change in a given predictor, holding all other variables constant. 95% confidence intervals in brackets; * p < 0.10, ** p < 0.05, *** p < 0.01. All p values were adjusted for multiple hypothesis tests using Holm’s method [107]. Note the 95% CIs were not adjusted for multiple hypothesis tests.
(DOCX)

S9 Table Weighted multinomial logistic regression results predicting class membership in the *UCA study* using UAS *survey weights.* Reported coefficients are average marginal effects (AMEs), representing the average change in the predicted probability of each outcome category associated with a one-unit change in a given predictor, holding all other variables constant. 95% confidence intervals in brackets; * p < 0.10, ** p < 0.05, *** p < 0.01. All p values were adjusted for multiple hypothesis tests using Holm’s method [107]. Note the 95% CIs were not adjusted for multiple hypothesis tests.
(DOCX)
