# Supplementary figures and images for: Personality traits, panel tenure, survey topic, and context as predictors of survey nonresponse patterns in high-frequency online longitudinal surveys
Source: PLoS One. 2025 Sep 22;20(9):e0332902. doi: 10.1371/journal.pone.0332902 (PMC12453192; doi:10.1371/journal.pone.0332902)

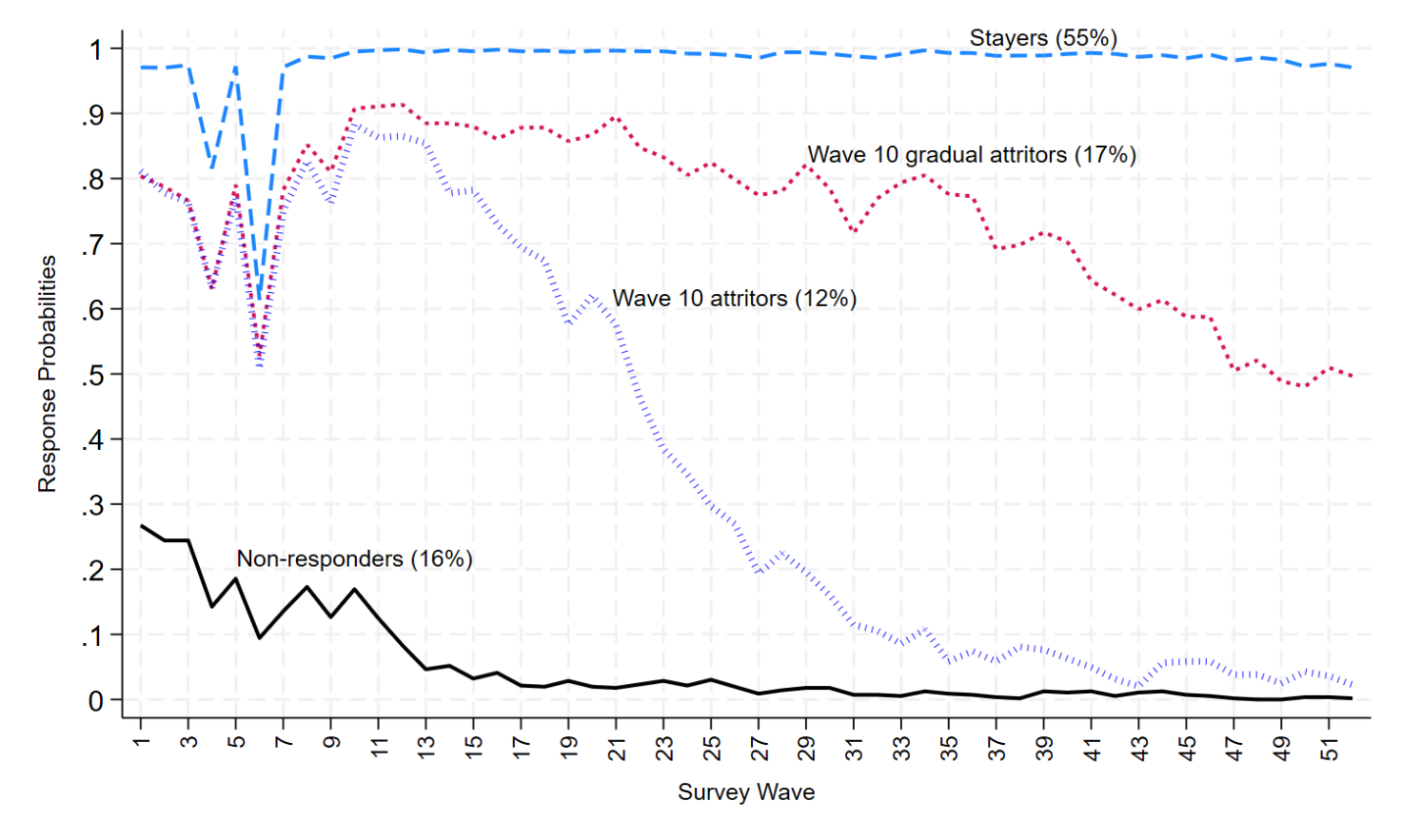

Supplement: S1 Fig — (TIF) [file pone.0332902.s001.tif]

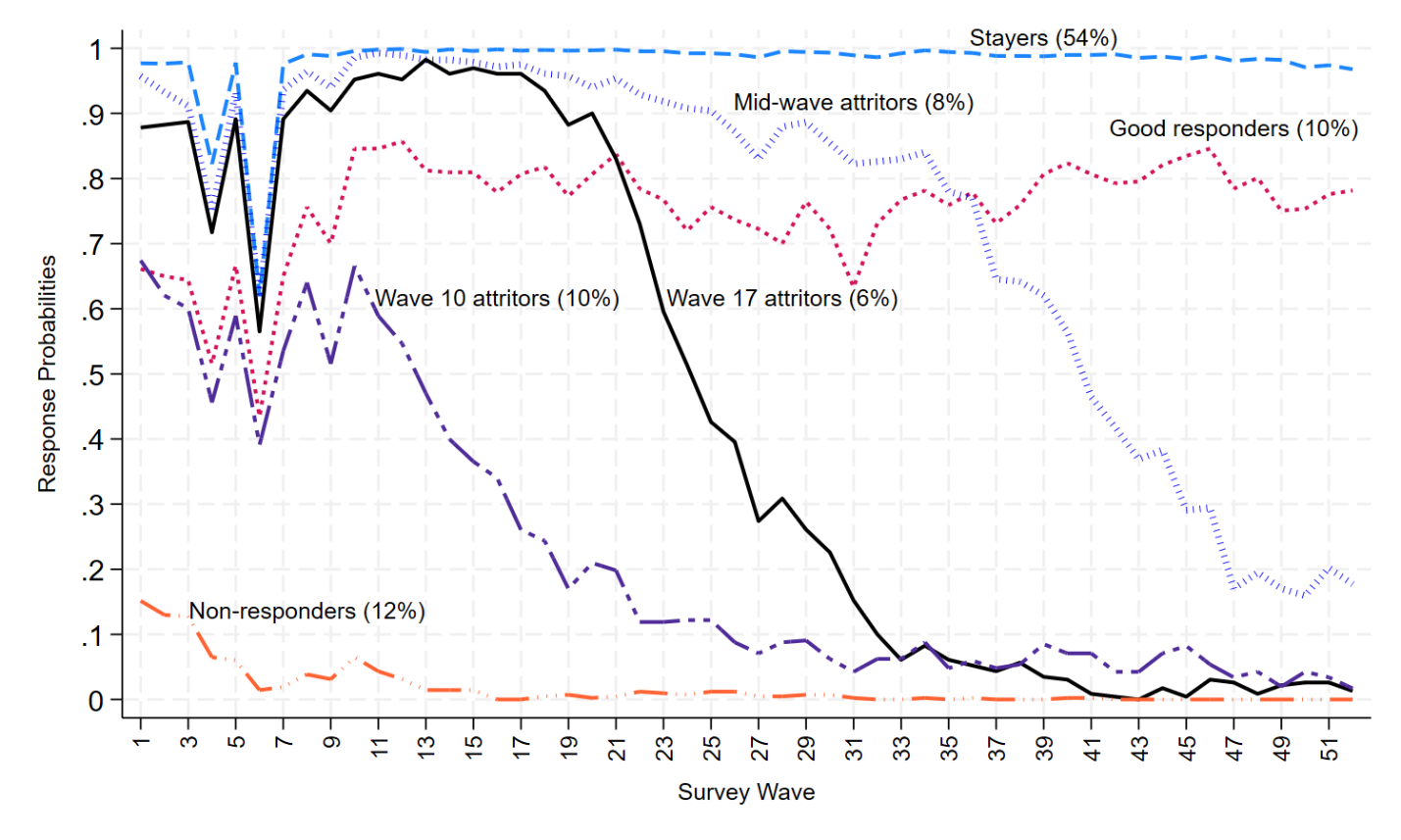

Supplement: S2 Fig — (TIF) [file pone.0332902.s002.tif]

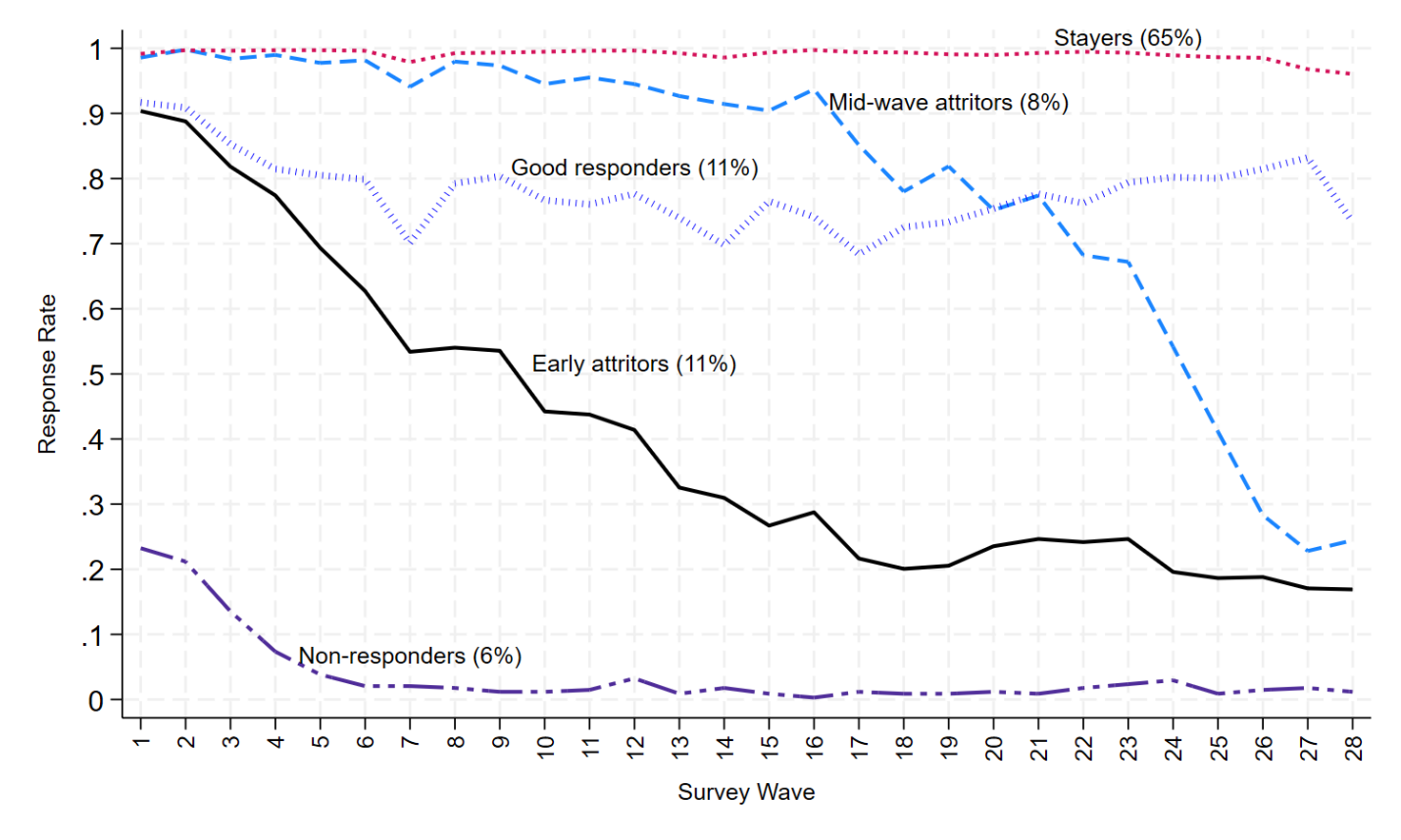

Supplement: S3 Fig — (TIF) [file pone.0332902.s003.tif]

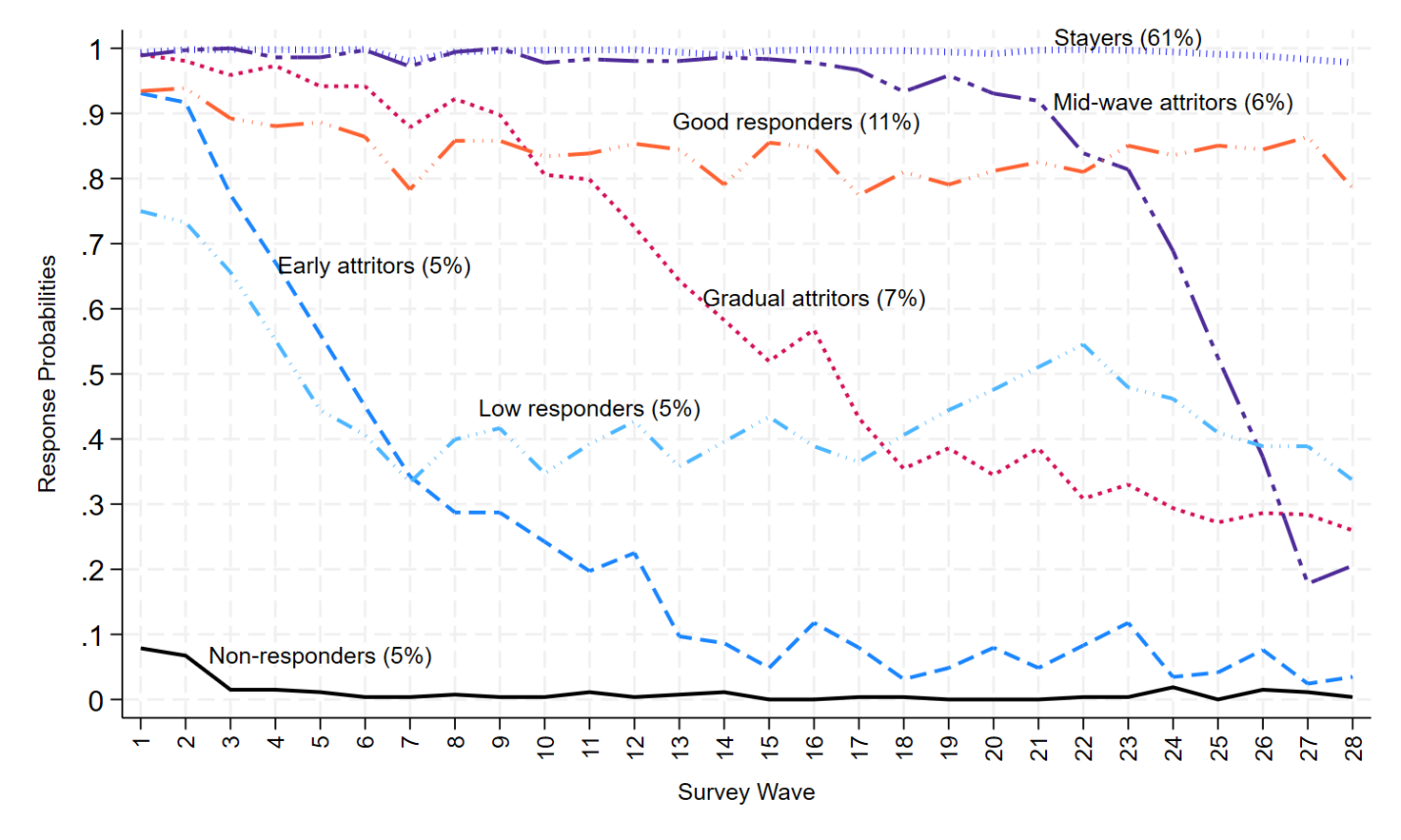

Supplement: S4 Fig — (TIF) [file pone.0332902.s004.tif]
