# Supplementary material for: Personality traits, panel tenure, survey topic, and context as predictors of survey nonresponse patterns in high-frequency online longitudinal surveys
Source: PLoS One. 2025 Sep 22;20(9):e0332902. doi: 10.1371/journal.pone.0332902 (PMC12453192; doi:10.1371/journal.pone.0332902)
Supplement: S2 Table — Reported coefficients are average marginal effects (AMEs), representing the average change in the predicted probability of each outcome category associated with a one-unit change in a given predictor variable, holding all other variables constant. 95% confidence intervals in brackets; * p < 0.10, ** p < 0.05, *** p < 0.01. All p values were adjusted for multiple hypothesis tests using Holm’s method [107]. Note the 95% CIs were not adjusted for multiple hypothesis tests. (DOCX) [file pone.0332902.s006.docx]

**S2 Table. Weighted multinomial logistic regressions predicting class membership in the *COVID-19* *panel study* using *probabilities predicting class membership for the chosen classes as weights*. Reported coefficients are average marginal effects (AMEs), representing the average change in the predicted probability of each outcome category associated with a one-unit change in a given predictor variable, holding all other variables constant.**

|  | Non-responders | Early attritors | Gradual attritors | Mid-wave attritors | Good responders | Stayers |
| --- | --- | --- | --- | --- | --- | --- |
| ***Big-5 Personality Traits*** |  |  |  |  |  |  |
| Conscientiousness Score | -0.000 | -0.001 | -0.002*** | -0.000 | -0.003*** | 0.008*** |
|  | [-0.001,0.001] | [-0.003,-0.000] | [-0.004,-0.001] | [-0.002,0.001] | [-0.005,-0.001] | [0.005,0.010] |
| Openness Score | 0.001 | 0.002*** | 0.001 | 0.001 | -0.000 | -0.005*** |
|  | [-0.000,0.002] | [0.001,0.003] | [-0.000,0.002] | [0.000,0.002] | [-0.001,0.001] | [-0.007,-0.003] |
| Extroversion Score | -0.000 | 0.002** | 0.001 | 0.000 | 0.001 | -0.003** |
|  | [-0.001,0.001] | [0.001,0.003] | [-0.001,0.002] | [-0.001,0.001] | [-0.001,0.002] | [-0.005,-0.001] |
| Neuroticism Score | 0.000 | 0.001 | 0.001 | 0.000 | 0.001 | -0.004** |
|  | [-0.001,0.001] | [0.000,0.003] | [-0.001,0.002] | [-0.001,0.002] | [-0.000,0.002] | [-0.006,-0.002] |
| Agreeableness Score | -0.000 | 0.001 | 0.000 | -0.001 | 0.002** | -0.003 |
|  | [-0.001,0.001] | [-0.000,0.003] | [-0.001,0.001] | [-0.002,0.001] | [0.001,0.004] | [-0.005,-0.000] |
| ***Panel Tenure***  ***(Ref: Less than 1 year)*** |  |  |  |  |  |  |
| 1 year and above | 0.046*** | -0.012 | -0.004 | 0.021* | -0.033** | -0.019 |
|  | [0.037,0.055] | [-0.027,0.004] | [-0.020,0.012] | [0.007,0.036] | [-0.053,-0.014] | [-0.047,0.010] |
| ***Hispanic***  ***(Ref: No)*** |  |  |  |  |  |  |
| Yes | 0.093*** | 0.004 | -0.002 | -0.016 | 0.035* | -0.113*** |
|  | [0.065,0.121] | [-0.013,0.022] | [-0.020,0.015] | [-0.034,0.001] | [0.011,0.059] | [-0.151,-0.076] |
| ***Race & Ethnicity***  ***(Ref: White only)*** |  |  |  |  |  |  |
| Black only | 0.020 | 0.032 | -0.016 | -0.012 | 0.028 | -0.052 |
|  | [-0.005,0.045] | [0.005,0.060] | [-0.039,0.007] | [-0.035,0.011] | [-0.003,0.060] | [-0.098,-0.007] |
| Others | 0.015 | -0.002 | -0.012 | -0.012 | 0.009 | 0.003 |
|  | [-0.002,0.032] | [-0.020,0.016] | [-0.030,0.007] | [-0.031,0.007] | [-0.014,0.031] | [-0.033,0.038] |
| ***Gender***  ***(Ref: Female)*** |  |  |  |  |  |  |
| Male | -0.016* | 0.013 | -0.001 | -0.002 | 0.008 | -0.002 |
|  | [-0.027,-0.005] | [-0.002,0.027] | [-0.015,0.014] | [-0.016,0.013] | [-0.009,0.025] | [-0.028,0.023] |
| ***Age Group***  ***(Ref: 18-44)*** |  |  |  |  |  |  |
| 45-64 | -0.009 | -0.052*** | -0.059*** | -0.024 | -0.032** | 0.175*** |
|  | [-0.021,0.004] | [-0.069,-0.035] | [-0.077,-0.041] | [-0.042,-0.006] | [-0.051,-0.012] | [0.144,0.206] |
| 65+ | 0.004 | -0.061*** | -0.080*** | -0.040*** | -0.042** | 0.219*** |
|  | [-0.016,0.024] | [-0.082,-0.041] | [-0.100,-0.059] | [-0.062,-0.019] | [-0.067,-0.016] | [0.179,0.260] |
| ***Education***  ***(Ref: GED or high school)*** |  |  |  |  |  |  |
| Some College | -0.012 | 0.008 | -0.026 | 0.003 | 0.005 | 0.022 |
|  | [-0.027,0.003] | [-0.011,0.026] | [-0.046,-0.005] | [-0.017,0.022] | [-0.016,0.026] | [-0.011,0.056] |
| College and above | -0.010 | -0.009 | -0.032* | -0.018 | -0.006 | 0.075*** |
|  | [-0.027,0.007] | [-0.028,0.010] | [-0.053,-0.010] | [-0.038,0.002] | [-0.029,0.016] | [0.039,0.111] |
| ***HH Income***  ***(Ref: Below $50K)*** |  |  |  |  |  |  |
| $50-$75K | -0.003 | -0.007 | -0.013 | 0.001 | 0.009 | 0.012 |
|  | [-0.017,0.011] | [-0.026,0.013] | [-0.032,0.006] | [-0.018,0.020] | [-0.014,0.031] | [-0.022,0.046] |
| $75K and above | 0.009 | -0.018 | -0.007 | -0.002 | -0.012 | 0.030 |
|  | [-0.006,0.024] | [-0.036,-0.001] | [-0.025,0.011] | [-0.020,0.016] | [-0.032,0.009] | [-0.002,0.062] |
| ***Employment Status***  ***(Ref: Currently working)*** |  |  |  |  |  |  |
| Currently not working | 0.010 | -0.020 | -0.010 | 0.001 | -0.017 | 0.035 |
|  | [-0.003,0.023] | [-0.035,-0.005] | [-0.025,0.006] | [-0.015,0.017] | [-0.035,0.001] | [0.007,0.063] |
| ***Household Size***  ***(Ref: 1)*** |  |  |  |  |  |  |
| 2 | -0.010 | -0.001 | 0.007 | 0.002 | 0.019 | -0.016 |
|  | [-0.026,0.006] | [-0.020,0.017] | [-0.013,0.026] | [-0.018,0.021] | [-0.002,0.040] | [-0.051,0.018] |
| 3 and above | -0.002 | 0.010 | 0.019 | 0.004 | 0.027 | -0.058** |
|  | [-0.020,0.015] | [-0.009,0.030] | [-0.001,0.039] | [-0.016,0.025] | [0.005,0.049] | [-0.094,-0.022] |
| ***Health Status*** |  |  |  |  |  |  |
| Self-report of health | -0.004 | -0.004 | -0.001 | 0.005 | -0.000 | 0.004 |
|  | [-0.010,0.002] | [-0.012,0.003] | [-0.009,0.007] | [-0.002,0.013] | [-0.009,0.009] | [-0.010,0.018] |
| n | 5,743 | | | | | |

95% confidence intervals in brackets; * p < 0.10, ** p < 0.05, *** p < 0.01. All p values were adjusted for multiple hypothesis tests using Holm’s method. Note the 95% CIs were not adjusted for multiple hypothesis tests.
