# Supplementary material for: Personality traits, panel tenure, survey topic, and context as predictors of survey nonresponse patterns in high-frequency online longitudinal surveys
Source: PLoS One. 2025 Sep 22;20(9):e0332902. doi: 10.1371/journal.pone.0332902 (PMC12453192; doi:10.1371/journal.pone.0332902)
Supplement: S3 Table — Reported coefficients are average marginal effects (AMEs), representing the average change in the predicted probability of each outcome category associated with a one-unit change in a given predictor variable, holding all other variables constant. 95% confidence intervals in brackets; * p < 0.10, ** p < 0.05, *** p < 0.01. All p values were adjusted for multiple hypothesis tests using Holm’s method [107]. Note the 95% CIs were not adjusted for multiple hypothesis tests. (DOCX) [file pone.0332902.s007.docx]

**S3 Table. Multinomial logistic regressions predicting class membership in the m*onthly events* *panel study* for the latent class model that is *one class smaller* than the chosen number of latent classes. Reported coefficients are average marginal effects (AMEs), representing the average change in the predicted probability of each outcome category associated with a one-unit change in a given predictor variable, holding all other variables constant.**

|  | Non-responders | Wave 10 attritors | Wave 10 gradual attritors | Stayers |
| --- | --- | --- | --- | --- |
| ***Big-5 Personality Traits*** |  |  |  |  |
| Conscientiousness Score | -0.003 | -0.001 | -0.004** | 0.008*** |
|  | [-0.005,-0.000] | [-0.004,0.001] | [-0.007,-0.002] | [0.005,0.011] |
| Openness Score | 0.002 | 0.001 | 0.000 | -0.003 |
|  | [-0.000,0.004] | [-0.001,0.003] | [-0.002,0.003] | [-0.006,-0.000] |
| Extroversion Score | 0.004*** | 0.000 | 0.001 | -0.005** |
|  | [0.002,0.006] | [-0.002,0.002] | [-0.002,0.003] | [-0.007,-0.002] |
| Neuroticism Score | 0.003* | 0.002 | 0.000 | -0.005** |
|  | [0.001,0.005] | [-0.000,0.004] | [-0.002,0.002] | [-0.008,-0.002] |
| Agreeableness Score | 0.002 | 0.001 | 0.003 | -0.006** |
|  | [-0.000,0.004] | [-0.001,0.004] | [0.000,0.006] | [-0.010,-0.003] |
| ***Panel Tenure***  ***(Ref: Less than 1 year)*** |  |  |  |  |
| 1 year and above | -0.034 | -0.007 | -0.010 | 0.052 |
|  | [-0.072,0.003] | [-0.042,0.029] | [-0.051,0.030] | [0.000,0.103] |
| ***Hispanic***  ***(Ref: No)*** |  |  |  |  |
| Yes | 0.051 | 0.004 | -0.029 | -0.026 |
|  | [-0.002,0.105] | [-0.044,0.051] | [-0.078,0.021] | [-0.097,0.044] |
| ***Race & Ethnicity***  ***(Ref: White only)*** |  |  |  |  |
| Black only | 0.017 | -0.018 | 0.052 | -0.051 |
|  | [-0.029,0.064] | [-0.059,0.023] | [-0.001,0.105] | [-0.116,0.013] |
| Others | 0.021 | -0.026 | 0.030 | -0.025 |
|  | [-0.020,0.062] | [-0.061,0.010] | [-0.016,0.076] | [-0.083,0.032] |
| ***Gender***  ***(Ref: Female)*** |  |  |  |  |
| Male | 0.027 | 0.007 | -0.007 | -0.027 |
|  | [0.003,0.051] | [-0.017,0.030] | [-0.034,0.020] | [-0.062,0.007] |
| ***Age Group***  ***(Ref: 50-64)*** |  |  |  |  |
| 65 and above | 0.016 | -0.003 | -0.016 | 0.003 |
|  | [-0.011,0.043] | [-0.029,0.023] | [-0.046,0.014] | [-0.036,0.042] |
| ***Education***  ***(Ref: GED or high school)*** |  |  |  |  |
| Some College | -0.013 | 0.002 | -0.017 | 0.029 |
|  | [-0.045,0.018] | [-0.027,0.030] | [-0.051,0.017] | [-0.015,0.072] |
| College and above | -0.039 | 0.005 | -0.030 | 0.063 |
|  | [-0.073,-0.005] | [-0.027,0.038] | [-0.067,0.008] | [0.015,0.112] |
| ***HH Income***  ***(Ref: Below $50K)*** |  |  |  |  |
| $50-$75K | -0.006 | 0.004 | -0.009 | 0.010 |
|  | [-0.037,0.026] | [-0.028,0.037] | [-0.044,0.027] | [-0.036,0.056] |
| $75K and above | -0.006 | -0.021 | -0.005 | 0.032 |
|  | [-0.037,0.024] | [-0.050,0.009] | [-0.040,0.029] | [-0.012,0.076] |
| ***Employment Status***  ***(Ref: Currently working)*** |  |  |  |  |
| Currently not working | -0.001 | -0.000 | -0.031 | 0.032 |
|  | [-0.028,0.025] | [-0.027,0.026] | [-0.062,-0.000] | [-0.007,0.071] |
| ***Household Size***  ***(Ref: 1)*** |  |  |  |  |
| 2 | -0.022 | 0.002 | 0.009 | 0.011 |
|  | [-0.052,0.008] | [-0.026,0.031] | [-0.024,0.042] | [-0.032,0.054] |
| 3 and above | 0.001 | 0.020 | 0.020 | -0.041 |
|  | [-0.035,0.036] | [-0.014,0.054] | [-0.018,0.058] | [-0.091,0.009] |
| ***Health Status*** |  |  |  |  |
|  | -0.008 | 0.014 | 0.024** | -0.030** |
|  | [-0.021,0.005] | [0.001,0.026] | [0.010,0.039] | [-0.049,-0.011] |
| n | 3446 | | | |

95% confidence intervals in brackets; * p < 0.10, ** p < 0.05, *** p < 0.01. All p values were adjusted for multiple hypothesis tests using Holm’s method. Note the 95% CIs were not adjusted for multiple hypothesis tests.
