# Supplementary material for: Personality traits, panel tenure, survey topic, and context as predictors of survey nonresponse patterns in high-frequency online longitudinal surveys
Source: PLoS One. 2025 Sep 22;20(9):e0332902. doi: 10.1371/journal.pone.0332902 (PMC12453192; doi:10.1371/journal.pone.0332902)
Supplement: S4 Table — Reported coefficients are average marginal effects (AMEs), representing the average change in the predicted probability of each outcome category associated with a one-unit change in a given predictor variable, holding all other variables constant. 95% confidence intervals in brackets; * p < 0.10, ** p < 0.05, *** p < 0.01. All p values were adjusted for multiple hypothesis tests using Holm’s method [107]. Note the 95% CIs were not adjusted for multiple hypothesis tests. (DOCX) [file pone.0332902.s008.docx]

**S4 Table. Multinomial logistic regressions predicting class membership in the *monthly events* *panel study* for the latent class model that is *one class larger* than the chosen number of latent classes. Reported coefficients are average marginal effects (AMEs), representing the average change in the predicted probability of each outcome category associated with a one-unit change in a given predictor variable, holding all other variables constant.**

|  | Non-responders | Wave 10 attritors | Wave 17 attritors | Mid-wave attritors | Good responders | Stayers |
| --- | --- | --- | --- | --- | --- | --- |
| ***Big-5 Personality Traits*** |  |  |  |  |  |  |
| Conscientiousness Score | -0.002 | -0.002 | 0.001 | -0.002 | -0.001 | 0.007*** |
|  | [-0.004,0.000] | [-0.004,-0.000] | [-0.001,0.003] | [-0.004,-0.001] | [-0.004,0.001] | [0.004,0.011] |
| Openness Score | 0.001 | 0.001 | 0.001 | -0.000 | 0.000 | -0.003 |
|  | [-0.001,0.002] | [-0.000,0.003] | [-0.001,0.002] | [-0.002,0.001] | [-0.001,0.002] | [-0.006,-0.000] |
| Extroversion Score | 0.004*** | 0.001 | 0.000 | -0.000 | 0.000 | -0.004* |
|  | [0.002,0.005] | [-0.001,0.002] | [-0.001,0.001] | [-0.002,0.001] | [-0.002,0.002] | [-0.007,-0.001] |
| Neuroticism Score | 0.002 | 0.002 | 0.001 | -0.001 | 0.000 | -0.005* |
|  | [0.000,0.004] | [0.000,0.004] | [-0.001,0.002] | [-0.002,0.001] | [-0.002,0.002] | [-0.008,-0.002] |
| Agreeableness Score | 0.001 | 0.002 | -0.000 | 0.001 | 0.002 | -0.006** |
|  | [-0.001,0.003] | [0.000,0.004] | [-0.002,0.001] | [-0.001,0.003] | [0.000,0.005] | [-0.010,-0.003] |
| ***Panel Tenure***  ***(Ref: Less than 1 year)*** |  |  |  |  |  |  |
| 1 year and above | -0.023 | -0.012 | -0.008 | 0.000 | -0.018 | 0.061 |
|  | [-0.056,0.009] | [-0.044,0.020] | [-0.035,0.019] | [-0.029,0.029] | [-0.051,0.016] | [0.009,0.113] |
| ***Hispanic***  ***(Ref: No)*** |  |  |  |  |  |  |
| Yes | 0.031 | 0.030 | -0.003 | -0.039 | 0.005 | -0.025 |
|  | [-0.015,0.077] | [-0.016,0.077] | [-0.039,0.032] | [-0.069,-0.009] | [-0.037,0.047] | [-0.095,0.046] |
| ***Race & Ethnicity***  ***(Ref: White only)*** |  |  |  |  |  |  |
| Black only | 0.011 | 0.013 | -0.021 | 0.014 | 0.037 | -0.055 |
|  | [-0.029,0.050] | [-0.027,0.053] | [-0.049,0.008] | [-0.024,0.052] | [-0.005,0.080] | [-0.119,0.010] |
| Others | 0.006 | 0.017 | -0.025 | -0.014 | 0.041 | -0.025 |
|  | [-0.028,0.040] | [-0.019,0.053] | [-0.049,-0.000] | [-0.044,0.016] | [0.002,0.080] | [-0.083,0.033] |
| ***Gender***  ***(Ref: Female)*** |  |  |  |  |  |  |
| Male | 0.008 | 0.031* | -0.002 | 0.000 | -0.011 | -0.026 |
|  | [-0.013,0.028] | [0.009,0.052] | [-0.019,0.016] | [-0.019,0.019] | [-0.032,0.011] | [-0.061,0.009] |
| ***Age Group***  ***(Ref: 50-64)*** |  |  |  |  |  |  |
| 65 and above | 0.016 | -0.015 | 0.011 | 0.013 | -0.022 | -0.003 |
|  | [-0.008,0.039] | [-0.038,0.009] | [-0.009,0.031] | [-0.010,0.035] | [-0.046,0.001] | [-0.042,0.037] |
| ***Education***  ***(Ref: GED or high school)*** |  |  |  |  |  |  |
| Some College | -0.013 | 0.007 | -0.002 | -0.010 | -0.004 | 0.023 |
|  | [-0.041,0.014] | [-0.019,0.033] | [-0.024,0.020] | [-0.035,0.014] | [-0.031,0.024] | [-0.021,0.066] |
| College and above | -0.031 | 0.004 | -0.005 | -0.013 | -0.020 | 0.065 |
|  | [-0.060,-0.001] | [-0.025,0.032] | [-0.030,0.019] | [-0.041,0.014] | [-0.049,0.010] | [0.016,0.114] |
| ***HH Income***  ***(Ref: Below $50K)*** |  |  |  |  |  |  |
| $50-$75K | -0.010 | -0.002 | 0.010 | 0.008 | -0.024 | 0.017 |
|  | [-0.037,0.016] | [-0.031,0.026] | [-0.014,0.035] | [-0.017,0.034] | [-0.051,0.004] | [-0.029,0.064] |
| $75K and above | -0.008 | -0.010 | -0.009 | 0.002 | -0.008 | 0.033 |
|  | [-0.034,0.018] | [-0.037,0.017] | [-0.031,0.013] | [-0.022,0.027] | [-0.036,0.019] | [-0.011,0.077] |
| ***Employment Status***  ***(Ref: Currently working)*** |  |  |  |  |  |  |
| Currently not working | -0.005 | -0.000 | 0.007 | -0.010 | -0.029 | 0.038 |
|  | [-0.028,0.018] | [-0.024,0.023] | [-0.013,0.026] | [-0.032,0.013] | [-0.054,-0.005] | [-0.002,0.077] |
| ***Household Size***  ***(Ref: 1)*** |  |  |  |  |  |  |
| 2 | -0.009 | -0.021 | 0.010 | 0.001 | 0.006 | 0.014 |
|  | [-0.034,0.016] | [-0.047,0.006] | [-0.011,0.031] | [-0.023,0.024] | [-0.021,0.032] | [-0.030,0.057] |
| 3 and above | -0.002 | 0.019 | -0.001 | 0.015 | 0.004 | -0.035 |
|  | [-0.031,0.028] | [-0.013,0.050] | [-0.025,0.023] | [-0.013,0.043] | [-0.026,0.033] | [-0.085,0.015] |
| ***Health Status*** |  |  |  |  |  |  |
|  | -0.012 | 0.005 | 0.011 | 0.016** | 0.004 | -0.025 |
|  | [-0.024,-0.001] | [-0.006,0.017] | [0.002,0.021] | [0.006,0.027] | [-0.008,0.016] | [-0.044,-0.006] |
| n | 3446 | | | | | |

95% confidence intervals in brackets; * p < 0.10, ** p < 0.05, *** p < 0.01. All p values were adjusted for multiple hypothesis tests using Holm’s method. Note the 95% CIs were not adjusted for multiple hypothesis tests.
