# Supplementary material for: Personality traits, panel tenure, survey topic, and context as predictors of survey nonresponse patterns in high-frequency online longitudinal surveys
Source: PLoS One. 2025 Sep 22;20(9):e0332902. doi: 10.1371/journal.pone.0332902 (PMC12453192; doi:10.1371/journal.pone.0332902)
Supplement: S5 Table — Reported coefficients are average marginal effects (AMEs), representing the average change in the predicted probability of each outcome category associated with a one-unit change in a given predictor variable, holding all other variables constant. 95% confidence intervals in brackets; * p < 0.10, ** p < 0.05, *** p < 0.01. All p values were adjusted for multiple hypothesis tests using Holm’s method [107]. Note the 95% CIs were not adjusted for multiple hypothesis tests. (DOCX) [file pone.0332902.s009.docx]

**S5 Table. Multinomial logistic regressions predicting class membership in the *COVID-19* *panel study* for the latent class model that is *one class smaller* than the chosen number of latent classes. Reported coefficients are average marginal effects (AMEs), representing the average change in the predicted probability of each outcome category associated with a one-unit change in a given predictor variable, holding all other variables constant.**

|  | Non-responders | Early attritors | Mid-wave attritors | Good responders | Stayers |
| --- | --- | --- | --- | --- | --- |
| ***Big-5 Personality Traits*** |  |  |  |  |  |
| Conscientiousness Score | -0.000 | -0.003*** | -0.001 | -0.004*** | 0.008*** |
|  | [-0.001,0.001] | [-0.005,-0.002] | [-0.002,0.001] | [-0.005,-0.002] | [0.005,0.010] |
| Openness Score | 0.001 | 0.003*** | 0.001 | -0.000 | -0.005*** |
|  | [0.000,0.002] | [0.001,0.004] | [-0.000,0.002] | [-0.002,0.001] | [-0.007,-0.002] |
| Extroversion Score | -0.000 | 0.002** | 0.001 | 0.001 | -0.003** |
|  | [-0.001,0.001] | [0.001,0.003] | [-0.000,0.002] | [-0.001,0.002] | [-0.005,-0.001] |
| Neuroticism Score | 0.001 | 0.001 | 0.001 | 0.001 | -0.004** |
|  | [-0.000,0.002] | [-0.000,0.003] | [-0.000,0.002] | [-0.001,0.002] | [-0.006,-0.002] |
| Agreeableness Score | -0.000 | 0.002 | -0.000 | 0.002 | -0.003 |
|  | [-0.001,0.001] | [-0.000,0.003] | [-0.001,0.001] | [0.000,0.003] | [-0.005,-0.001] |
| ***Panel Tenure***  ***(Ref: Less than 1 year)*** |  |  |  |  |  |
| 1 year and above | 0.035*** | -0.007 | 0.028*** | -0.037*** | -0.019 |
|  | [0.023,0.047] | [-0.026,0.011] | [0.013,0.044] | [-0.057,-0.016] | [-0.047,0.009] |
| ***Hispanic***  ***(Ref: No)*** |  |  |  |  |  |
| Yes | 0.080*** | 0.005 | -0.005 | 0.030 | -0.109*** |
|  | [0.053,0.106] | [-0.017,0.026] | [-0.025,0.014] | [0.006,0.054] | [-0.146,-0.072] |
| ***Race & Ethnicity***  ***(Ref: White only)*** |  |  |  |  |  |
| Black only | 0.018 | 0.032 | -0.017 | 0.015 | -0.048 |
|  | [-0.008,0.044] | [0.000,0.063] | [-0.041,0.008] | [-0.016,0.047] | [-0.094,-0.003] |
| Others | 0.009 | 0.003 | -0.013 | 0.004 | -0.003 |
|  | [-0.009,0.027] | [-0.020,0.025] | [-0.033,0.008] | [-0.019,0.027] | [-0.038,0.032] |
| ***Gender***  ***(Ref: Female)*** |  |  |  |  |  |
| Male | -0.008 | 0.010 | -0.004 | 0.003 | -0.001 |
|  | [-0.021,0.004] | [-0.007,0.027] | [-0.019,0.012] | [-0.014,0.020] | [-0.026,0.024] |
| ***Age Group***  ***(Ref: 18-44)*** |  |  |  |  |  |
| 45-64 | -0.014 | -0.084*** | -0.039*** | -0.039*** | 0.176*** |
|  | [-0.028,0.001] | [-0.104,-0.063] | [-0.058,-0.020] | [-0.059,-0.019] | [0.145,0.206] |
| 65+ | -0.005 | -0.104*** | -0.060*** | -0.045** | 0.214*** |
|  | [-0.026,0.017] | [-0.128,-0.080] | [-0.083,-0.037] | [-0.072,-0.018] | [0.173,0.254] |
| ***Education***  ***(Ref: GED or high school)*** |  |  |  |  |  |
| Some College | -0.010 | -0.005 | -0.011 | -0.002 | 0.028 |
|  | [-0.027,0.006] | [-0.027,0.018] | [-0.033,0.010] | [-0.024,0.020] | [-0.005,0.061] |
| College and above | -0.009 | -0.023 | -0.033* | -0.012 | 0.078*** |
|  | [-0.028,0.009] | [-0.047,0.001] | [-0.056,-0.011] | [-0.036,0.011] | [0.042,0.114] |
| ***HH Income***  ***(Ref: Below $50K)*** |  |  |  |  |  |
| $50-$75K | -0.010 | -0.013 | 0.004 | 0.005 | 0.014 |
|  | [-0.026,0.006] | [-0.036,0.010] | [-0.016,0.024] | [-0.019,0.028] | [-0.019,0.048] |
| $75K and above | -0.004 | -0.017 | 0.009 | -0.015 | 0.027 |
|  | [-0.020,0.013] | [-0.038,0.004] | [-0.010,0.029] | [-0.036,0.006] | [-0.005,0.059] |
| ***Employment Status***  ***(Ref: Currently working)*** |  |  |  |  |  |
| Currently not working | 0.003 | -0.019 | -0.006 | -0.016 | 0.038* |
|  | [-0.011,0.017] | [-0.037,-0.001] | [-0.023,0.011] | [-0.035,0.003] | [0.009,0.066] |
| ***Household Size***  ***(Ref: 1)*** |  |  |  |  |  |
| 2 | -0.007 | -0.001 | 0.010 | 0.015 | -0.017 |
|  | [-0.025,0.010] | [-0.023,0.022] | [-0.012,0.031] | [-0.007,0.038] | [-0.052,0.017] |
| 3 and above | 0.002 | 0.018 | 0.009 | 0.027 | -0.057** |
|  | [-0.016,0.021] | [-0.005,0.042] | [-0.012,0.031] | [0.004,0.050] | [-0.093,-0.021] |
| ***Health Status*** |  |  |  |  |  |
| Self-report of health | -0.005 | -0.005 | 0.006 | -0.002 | 0.006 |
|  | [-0.012,0.002] | [-0.014,0.004] | [-0.002,0.015] | [-0.012,0.007] | [-0.008,0.020] |
| n | 5743 | | | | |

95% confidence intervals in brackets; * p < 0.10, ** p < 0.05, *** p < 0.01. All p values were adjusted for multiple hypothesis tests using Holm’s method. Note the 95% CIs were not adjusted for multiple hypothesis tests.
