# Supplementary material for: Personality traits, panel tenure, survey topic, and context as predictors of survey nonresponse patterns in high-frequency online longitudinal surveys
Source: PLoS One. 2025 Sep 22;20(9):e0332902. doi: 10.1371/journal.pone.0332902 (PMC12453192; doi:10.1371/journal.pone.0332902)
Supplement: S6 Table — Reported coefficients are average marginal effects (AMEs), representing the average change in the predicted probability of each outcome category associated with a one-unit change in a given predictor variable, holding all other variables constant. 95% confidence intervals in brackets; * p < 0.10, ** p < 0.05, *** p < 0.01. All p values were adjusted for multiple hypothesis tests using Holm’s method [107]. Note the 95% CIs were not adjusted for multiple hypothesis tests. (DOCX) [file pone.0332902.s010.docx]

**S6 Table. Multinomial logistic regressions predicting class membership in the *COVID-19* *panel study* for the latent class model that is *one class larger* than the chosen number of latent classes. Reported coefficients are average marginal effects (AMEs), representing the average change in the predicted probability of each outcome category associated with a one-unit change in a given predictor variable, holding all other variables constant.**

|  | Non-responders | Early attritors | Low Responders | Gradual attritors | Mid-wave attritors | Good responders | Stayers |
| --- | --- | --- | --- | --- | --- | --- | --- |
| ***Big-5 Personality Traits*** |  |  |  |  |  |  |  |
| Conscientiousness Score | -0.000 | -0.001 | -0.002*** | -0.002** | -0.000 | -0.002 | 0.008*** |
|  | [-0.001,0.001] | [-0.002,0.000] | [-0.003,-0.001] | [-0.003,-0.001] | [-0.002,0.001] | [-0.004,-0.001] | [0.005,0.010] |
| Openness Score | 0.001 | 0.001 | 0.001 | 0.001 | 0.001 | 0.000 | -0.005*** |
|  | [-0.000,0.002] | [0.000,0.002] | [0.000,0.002] | [-0.000,0.002] | [-0.000,0.002] | [-0.001,0.002] | [-0.007,-0.003] |
| Extroversion Score | -0.000 | 0.001* | 0.001 | 0.001 | 0.000 | 0.000 | -0.003** |
|  | [-0.001,0.001] | [0.000,0.002] | [0.000,0.002] | [-0.001,0.002] | [-0.001,0.001] | [-0.001,0.002] | [-0.005,-0.001] |
| Neuroticism Score | 0.000 | 0.001 | 0.001 | 0.000 | 0.000 | 0.001 | -0.004*** |
|  | [-0.001,0.001] | [-0.000,0.002] | [-0.000,0.002] | [-0.001,0.002] | [-0.001,0.001] | [-0.000,0.003] | [-0.006,-0.002] |
| Agreeableness Score | -0.000 | 0.001 | 0.002 | -0.000 | -0.001 | 0.002 | -0.003 |
|  | [-0.001,0.001] | [-0.001,0.002] | [0.000,0.003] | [-0.002,0.001] | [-0.002,0.001] | [0.000,0.004] | [-0.005,-0.001] |
| ***Panel Tenure***  ***(Ref: Less than 1 year)*** |  |  |  |  |  |  |  |
| 1 year and above | 0.044*** | -0.004 | -0.015 | -0.005 | 0.020* | -0.025 | -0.015 |
|  | [0.034,0.053] | [-0.017,0.009] | [-0.029,-0.001] | [-0.020,0.011] | [0.006,0.034] | [-0.045,-0.005] | [-0.044,0.014] |
| ***Hispanic***  ***(Ref: No)*** |  |  |  |  |  |  |  |
| Yes | 0.088*** | 0.001 | 0.008 | -0.003 | -0.016 | 0.041** | -0.119*** |
|  | [0.061,0.116] | [-0.014,0.016] | [-0.008,0.023] | [-0.020,0.014] | [-0.033,-0.000] | [0.016,0.067] | [-0.157,-0.081] |
| ***Race & Ethnicity***  ***(Ref: White only)*** |  |  |  |  |  |  |  |
| Black only | 0.018 | 0.015 | 0.035 | -0.023 | -0.015 | 0.024 | -0.054 |
|  | [-0.007,0.042] | [-0.008,0.038] | [0.010,0.061] | [-0.044,-0.003] | [-0.036,0.006] | [-0.009,0.058] | [-0.100,-0.008] |
| Others | 0.016 | -0.005 | -0.002 | -0.010 | -0.015 | 0.015 | -0.000 |
|  | [-0.001,0.034] | [-0.020,0.010] | [-0.017,0.013] | [-0.028,0.008] | [-0.033,0.002] | [-0.009,0.040] | [-0.036,0.036] |
| ***Gender***  ***(Ref: Female)*** |  |  |  |  |  |  |  |
| Male | -0.014 | 0.016 | -0.001 | -0.008 | 0.001 | 0.004 | 0.001 |
|  | [-0.025,-0.003] | [0.004,0.029] | [-0.013,0.011] | [-0.022,0.005] | [-0.012,0.015] | [-0.014,0.022] | [-0.025,0.027] |
| ***Age Group***  ***(Ref: 18-44)*** |  |  |  |  |  |  |  |
| 45-64 | -0.009 | -0.038*** | -0.026*** | -0.055*** | -0.016 | -0.034** | 0.178*** |
|  | [-0.021,0.004] | [-0.052,-0.024] | [-0.041,-0.012] | [-0.072,-0.037] | [-0.033,0.001] | [-0.055,-0.014] | [0.147,0.209] |
| 65+ | 0.004 | -0.039*** | -0.036*** | -0.075*** | -0.030* | -0.041* | 0.218*** |
|  | [-0.016,0.024] | [-0.058,-0.021] | [-0.054,-0.019] | [-0.094,-0.055] | [-0.051,-0.010] | [-0.069,-0.013] | [0.176,0.259] |
| ***Education***  ***(Ref: GED or high school)*** |  |  |  |  |  |  |  |
| Some College | -0.010 | 0.008 | -0.006 | -0.029* | 0.008 | 0.006 | 0.023 |
|  | [-0.025,0.004] | [-0.007,0.023] | [-0.023,0.011] | [-0.049,-0.009] | [-0.010,0.026] | [-0.016,0.029] | [-0.010,0.057] |
| College and above | -0.008 | 0.001 | -0.027* | -0.034** | -0.012 | -0.001 | 0.080*** |
|  | [-0.025,0.008] | [-0.014,0.017] | [-0.044,-0.009] | [-0.055,-0.012] | [-0.030,0.006] | [-0.025,0.023] | [0.044,0.116] |
| ***HH Income***  ***(Ref: Below $50K)*** |  |  |  |  |  |  |  |
| $50-$75K | -0.005 | -0.007 | 0.004 | -0.006 | -0.009 | 0.018 | 0.004 |
|  | [-0.019,0.009] | [-0.023,0.010] | [-0.012,0.021] | [-0.024,0.012] | [-0.026,0.008] | [-0.006,0.043] | [-0.030,0.039] |
| $75K and above | 0.006 | -0.014 | -0.003 | -0.000 | -0.002 | -0.014 | 0.027 |
|  | [-0.008,0.021] | [-0.029,0.001] | [-0.018,0.012] | [-0.018,0.017] | [-0.019,0.015] | [-0.036,0.007] | [-0.005,0.060] |
| ***Employment Status***  ***(Ref: Currently working)*** |  |  |  |  |  |  |  |
| Currently not working | 0.009 | -0.014 | -0.010 | -0.010 | 0.002 | -0.017 | 0.039* |
|  | [-0.004,0.022] | [-0.027,-0.001] | [-0.023,0.003] | [-0.025,0.005] | [-0.013,0.017] | [-0.036,0.003] | [0.010,0.068] |
| ***Household Size***  ***(Ref: 1)*** |  |  |  |  |  |  |  |
| 2 | -0.009 | 0.004 | -0.003 | 0.004 | 0.008 | 0.010 | -0.015 |
|  | [-0.025,0.007] | [-0.011,0.020] | [-0.020,0.015] | [-0.015,0.024] | [-0.010,0.026] | [-0.013,0.033] | [-0.050,0.020] |
| 3 and above | -0.002 | 0.013 | -0.002 | 0.011 | 0.008 | 0.028 | -0.057** |
|  | [-0.019,0.015] | [-0.003,0.029] | [-0.019,0.015] | [-0.008,0.031] | [-0.010,0.027] | [0.004,0.052] | [-0.094,-0.020] |
| ***Health Status*** |  |  |  |  |  |  |  |
| Self-report of health | -0.004 | -0.001 | -0.005 | 0.001 | 0.006 | -0.001 | 0.005 |
|  | [-0.010,0.002] | [-0.008,0.005] | [-0.011,0.001] | [-0.007,0.008] | [-0.002,0.013] | [-0.010,0.009] | [-0.009,0.019] |
| n | 5,743 | | | | | | |

95% confidence intervals in brackets; * p < 0.10, ** p < 0.05, *** p < 0.01. All p values were adjusted for multiple hypothesis tests using Holm’s method. Note the 95% CIs were not adjusted for multiple hypothesis tests.
