# Supplementary material for: Personality traits, panel tenure, survey topic, and context as predictors of survey nonresponse patterns in high-frequency online longitudinal surveys
Source: PLoS One. 2025 Sep 22;20(9):e0332902. doi: 10.1371/journal.pone.0332902 (PMC12453192; doi:10.1371/journal.pone.0332902)
Supplement: S7 Table — Reported coefficients are average marginal effects (AMEs), representing the average change in the predicted probability of each outcome category associated with a one-unit change in a given predictor variable, holding all other variables constant. 95% confidence intervals in brackets; * p < 0.10, ** p < 0.05, *** p < 0.01. All p values were adjusted for multiple hypothesis tests using Holm’s method [107]. Note the 95% CIs were not adjusted for multiple hypothesis tests. (DOCX) [file pone.0332902.s011.docx]

**S7 Table. Multinomial logistic regressions predicting class membership in the *COVID-19 panel study*, restricted to respondents *aged 50 and above*. Reported coefficients are average marginal effects (AMEs), representing the average change in the predicted probability of each outcome category associated with a one-unit change in a given predictor variable, holding all other variables constant.**

|  | Non-responders | Early attritors | Gradual attritors | Mid-wave attritors | Good responders | Stayers |
| --- | --- | --- | --- | --- | --- | --- |
| ***Big-5 Personality Traits*** |  |  |  |  |  |  |
| Conscientiousness Score | -0.000 | -0.001 | -0.002 | 0.000 | -0.003** | 0.005** |
|  | [-0.002,0.001] | [-0.002,0.001] | [-0.003,-0.000] | [-0.001,0.002] | [-0.005,-0.001] | [0.002,0.008] |
| Openness Score | 0.000 | 0.001 | -0.000 | 0.001 | 0.001 | -0.003 |
|  | [-0.001,0.001] | [0.000,0.002] | [-0.002,0.001] | [-0.001,0.002] | [-0.001,0.003] | [-0.006,-0.000] |
| Extroversion Score | -0.001 | 0.001 | 0.001 | 0.000 | 0.000 | -0.001 |
|  | [-0.002,0.001] | [-0.001,0.002] | [-0.001,0.002] | [-0.001,0.002] | [-0.002,0.002] | [-0.003,0.002] |
| Neuroticism Score | -0.000 | 0.002 | 0.001 | 0.000 | 0.000 | -0.002 |
|  | [-0.001,0.001] | [0.000,0.003] | [-0.001,0.002] | [-0.001,0.002] | [-0.002,0.002] | [-0.005,0.000] |
| Agreeableness Score | -0.001 | 0.001 | 0.001 | -0.000 | 0.001 | -0.002 |
|  | [-0.002,0.001] | [-0.000,0.003] | [-0.001,0.002] | [-0.002,0.002] | [-0.001,0.003] | [-0.006,0.001] |
| ***Panel Tenure***  ***(Ref: Less than 1 year)*** |  |  |  |  |  |  |
| 1 year and above | 0.023*** | -0.010 | -0.007 | 0.033** | -0.038 | -0.001 |
|  | [0.010,0.036] | [-0.031,0.010] | [-0.028,0.014] | [0.013,0.053] | [-0.067,-0.009] | [-0.044,0.041] |
| ***Hispanic***  ***(Ref: No)*** |  |  |  |  |  |  |
| Yes | 0.037 | 0.017 | 0.015 | -0.017 | 0.041 | -0.092 |
|  | [-0.004,0.078] | [-0.014,0.047] | [-0.018,0.047] | [-0.048,0.013] | [-0.003,0.084] | [-0.160,-0.024] |
| ***Race & Ethnicity***  ***(Ref: White only)*** |  |  |  |  |  |  |
| Black only | 0.023 | 0.011 | 0.009 | -0.018 | 0.028 | -0.052 |
|  | [-0.011,0.058] | [-0.018,0.040] | [-0.021,0.039] | [-0.048,0.011] | [-0.014,0.069] | [-0.116,0.012] |
| Others | 0.011 | 0.004 | -0.015 | -0.026 | -0.008 | 0.033 |
|  | [-0.014,0.037] | [-0.020,0.028] | [-0.036,0.006] | [-0.052,0.000] | [-0.037,0.022] | [-0.018,0.084] |
| ***Gender***  ***(Ref: Female)*** |  |  |  |  |  |  |
| Male | -0.005 | 0.017 | 0.001 | -0.000 | 0.001 | -0.014 |
|  | [-0.019,0.008] | [0.002,0.032] | [-0.014,0.017] | [-0.019,0.018] | [-0.018,0.021] | [-0.047,0.019] |
| ***Age Group***  ***(Ref: 50-64)*** |  |  |  |  |  |  |
| 65+ | 0.008 | -0.008 | -0.012 | -0.013 | 0.001 | 0.024 |
|  | [-0.007,0.024] | [-0.024,0.008] | [-0.029,0.005] | [-0.033,0.007] | [-0.021,0.023] | [-0.013,0.060] |
| ***Education***  ***(Ref: GED or high school)*** |  |  |  |  |  |  |
| Some College | -0.012 | 0.005 | -0.021 | 0.006 | -0.007 | 0.029 |
|  | [-0.033,0.008] | [-0.013,0.022] | [-0.042,-0.000] | [-0.019,0.031] | [-0.033,0.019] | [-0.014,0.073] |
| College and above | -0.012 | 0.009 | -0.018 | -0.021 | -0.017 | 0.059 |
|  | [-0.034,0.009] | [-0.010,0.029] | [-0.041,0.005] | [-0.047,0.004] | [-0.045,0.011] | [0.012,0.106] |
| ***HH Income***  ***(Ref: Below $50K)*** |  |  |  |  |  |  |
| $50-$75K | 0.007 | -0.016 | -0.008 | 0.002 | 0.006 | 0.009 |
|  | [-0.010,0.024] | [-0.036,0.005] | [-0.030,0.013] | [-0.023,0.027] | [-0.021,0.032] | [-0.035,0.053] |
| $75K and above | 0.019 | -0.017 | -0.017 | -0.007 | -0.005 | 0.027 |
|  | [0.000,0.038] | [-0.036,0.002] | [-0.037,0.002] | [-0.030,0.016] | [-0.029,0.020] | [-0.015,0.069] |
| ***Employment Status***  ***(Ref: Currently working)*** |  |  |  |  |  |  |
| Currently not working | 0.014 | -0.004 | -0.014 | -0.008 | -0.019 | 0.032 |
|  | [-0.001,0.029] | [-0.020,0.012] | [-0.032,0.004] | [-0.029,0.013] | [-0.042,0.003] | [-0.005,0.069] |
| ***Household Size***  ***(Ref: 1)*** |  |  |  |  |  |  |
| 2 | -0.017 | 0.006 | 0.005 | -0.000 | 0.001 | 0.006 |
|  | [-0.036,0.003] | [-0.010,0.021] | [-0.013,0.023] | [-0.023,0.022] | [-0.023,0.024] | [-0.034,0.046] |
| 3 and above | -0.016 | 0.020 | 0.011 | 0.010 | 0.008 | -0.034 |
|  | [-0.038,0.006] | [-0.001,0.041] | [-0.010,0.033] | [-0.017,0.038] | [-0.020,0.036] | [-0.082,0.014] |
| ***Health Status*** |  |  |  |  |  |  |
| Self-report of health | -0.007 | 0.001 | 0.000 | 0.006 | 0.006 | -0.005 |
|  | [-0.015,0.001] | [-0.007,0.009] | [-0.009,0.009] | [-0.004,0.017] | [-0.005,0.017] | [-0.024,0.013] |
| n | 3045 | | | | | |

95% confidence intervals in brackets; * p < 0.10, ** p < 0.05, *** p < 0.01. All p values were adjusted for multiple hypothesis tests using Holm’s method. Note the 95% CIs were not adjusted for multiple hypothesis tests.
