# Supplementary material for: Personality traits, panel tenure, survey topic, and context as predictors of survey nonresponse patterns in high-frequency online longitudinal surveys
Source: PLoS One. 2025 Sep 22;20(9):e0332902. doi: 10.1371/journal.pone.0332902 (PMC12453192; doi:10.1371/journal.pone.0332902)
Supplement: S8 Table — Reported coefficients are average marginal effects (AMEs), representing the average change in the predicted probability of each outcome category associated with a one-unit change in a given predictor, holding all other variables constant. 95% confidence intervals in brackets; * p < 0.10, ** p < 0.05, *** p < 0.01. All p values were adjusted for multiple hypothesis tests using Holm’s method [107]. Note the 95% CIs were not adjusted for multiple hypothesis tests. (DOCX) [file pone.0332902.s012.docx]

**S8 Table. Weighted multinomial logistic regression results predicting class membership in the *monthly events panel study* using UAS *survey weights*. Reported coefficients are average marginal effects (AMEs), representing the average change in the predicted probability of each outcome category associated with a one-unit change in a given predictor, holding all other variables constant.**

|  | Non-responders | Wave 10 attritors | Mid-wave attritors | Good responders | Stayers |
| --- | --- | --- | --- | --- | --- |
| ***Big-5 Personality Traits*** |  |  |  |  |  |
| Conscientiousness Score | -0.004** | 0.000 | -0.002 | -0.001 | 0.007*** |
|  | [-0.006,-0.001] | [-0.002,0.002] | [-0.004,-0.000] | [-0.003,0.001] | [0.003,0.010] |
| Openness Score | 0.001 | 0.001 | 0.000 | 0.001 | -0.003 |
|  | [-0.001,0.003] | [-0.001,0.003] | [-0.001,0.002] | [-0.001,0.003] | [-0.006,-0.000] |
| Extroversion Score | 0.005*** | 0.001 | -0.000 | 0.001 | -0.007*** |
|  | [0.003,0.007] | [-0.001,0.003] | [-0.002,0.001] | [-0.001,0.003] | [-0.010,-0.004] |
| Neuroticism Score | 0.002 | 0.002 | -0.001 | 0.001 | -0.005** |
|  | [0.000,0.004] | [0.000,0.004] | [-0.002,0.001] | [-0.001,0.003] | [-0.008,-0.002] |
| Agreeableness Score | 0.002 | 0.001 | 0.000 | 0.002 | -0.006** |
|  | [-0.000,0.004] | [-0.001,0.004] | [-0.002,0.002] | [-0.000,0.004] | [-0.009,-0.002] |
| ***Panel Tenure***  ***(Ref: Less than 1 year)*** |  |  |  |  |  |
| 1 year and above | -0.004 | -0.022 | -0.006 | -0.030 | 0.063 |
|  | [-0.039,0.030] | [-0.060,0.016] | [-0.037,0.025] | [-0.068,0.008] | [0.010,0.116] |
| ***Hispanic***  ***(Ref: No)*** |  |  |  |  |  |
| Yes | 0.046 | 0.004 | -0.036 | 0.025 | -0.039 |
|  | [0.005,0.087] | [-0.034,0.042] | [-0.062,-0.010] | [-0.013,0.063] | [-0.095,0.017] |
| ***Race & Ethnicity***  ***(Ref: White only)*** |  |  |  |  |  |
| Black only | 0.005 | 0.016 | 0.015 | 0.026 | -0.062 |
|  | [-0.032,0.042] | [-0.024,0.056] | [-0.019,0.048] | [-0.013,0.066] | [-0.118,-0.005] |
| Others | 0.021 | 0.004 | -0.041** | 0.004 | 0.012 |
|  | [-0.020,0.061] | [-0.035,0.044] | [-0.066,-0.017] | [-0.033,0.041] | [-0.046,0.070] |
| ***Gender***  ***(Ref: Female)*** |  |  |  |  |  |
| Male | 0.006 | 0.013 | 0.015 | -0.000 | -0.034 |
|  | [-0.017,0.030] | [-0.011,0.037] | [-0.004,0.034] | [-0.024,0.023] | [-0.069,0.001] |
| ***Age Group***  ***(Ref: 50-64)*** |  |  |  |  |  |
| 65 and above | 0.019 | 0.002 | 0.010 | -0.030 | -0.001 |
|  | [-0.007,0.045] | [-0.025,0.029] | [-0.011,0.032] | [-0.055,-0.005] | [-0.041,0.038] |
| ***Education***  ***(Ref: GED or high school)*** |  |  |  |  |  |
| Some College | 0.009 | -0.011 | -0.021 | 0.002 | 0.021 |
|  | [-0.020,0.038] | [-0.040,0.018] | [-0.043,0.002] | [-0.027,0.030] | [-0.022,0.064] |
| College and above | -0.028 | -0.013 | -0.003 | -0.032 | 0.076** |
|  | [-0.057,0.000] | [-0.044,0.018] | [-0.029,0.023] | [-0.061,-0.003] | [0.031,0.122] |
| ***HH Income***  ***(Ref: Below $50K)*** |  |  |  |  |  |
| $50-$75K | -0.022 | 0.007 | 0.009 | -0.036 | 0.042 |
|  | [-0.051,0.008] | [-0.026,0.040] | [-0.017,0.035] | [-0.064,-0.008] | [-0.004,0.089] |
| $75K and above | -0.008 | -0.009 | 0.014 | 0.015 | -0.012 |
|  | [-0.038,0.022] | [-0.039,0.022] | [-0.012,0.040] | [-0.016,0.047] | [-0.058,0.034] |
| ***Employment Status***  ***(Ref: Currently working)*** |  |  |  |  |  |
| Currently not working | 0.008 | -0.019 | 0.005 | -0.017 | 0.022 |
|  | [-0.018,0.034] | [-0.047,0.009] | [-0.017,0.028] | [-0.043,0.010] | [-0.018,0.062] |
| ***Household Size***  ***(Ref: 1)*** |  |  |  |  |  |
| 2 | -0.015 | 0.003 | -0.014 | 0.019 | 0.008 |
|  | [-0.044,0.013] | [-0.026,0.032] | [-0.038,0.010] | [-0.009,0.047] | [-0.036,0.052] |
| 3 and above | -0.001 | 0.022 | 0.009 | 0.014 | -0.044 |
|  | [-0.035,0.033] | [-0.012,0.056] | [-0.020,0.038] | [-0.017,0.045] | [-0.094,0.007] |
| ***Health Status*** |  |  |  |  |  |
|  | -0.015 | 0.010 | 0.023*** | 0.012 | -0.030** |
|  | [-0.028,-0.002] | [-0.003,0.024] | [0.012,0.034] | [-0.001,0.025] | [-0.050,-0.011] |
| n | 3,360 | | | | |

95% confidence intervals in brackets; * p < 0.10, ** p < 0.05, *** p < 0.01. All p values were adjusted for multiple hypothesis tests using Holm’s method. Note the 95% CIs were not adjusted for multiple hypothesis tests.
