# Supplementary material for: Personality traits, panel tenure, survey topic, and context as predictors of survey nonresponse patterns in high-frequency online longitudinal surveys
Source: PLoS One. 2025 Sep 22;20(9):e0332902. doi: 10.1371/journal.pone.0332902 (PMC12453192; doi:10.1371/journal.pone.0332902)
Supplement: S9 Table — Reported coefficients are average marginal effects (AMEs), representing the average change in the predicted probability of each outcome category associated with a one-unit change in a given predictor, holding all other variables constant. 95% confidence intervals in brackets; * p < 0.10, ** p < 0.05, *** p < 0.01. All p values were adjusted for multiple hypothesis tests using Holm’s method [107]. Note the 95% CIs were not adjusted for multiple hypothesis tests. (DOCX) [file pone.0332902.s013.docx]

**S9 Table. Weighted multinomial logistic regression results predicting class membership in the *UCA study* using UAS *survey weights*. Reported coefficients are average marginal effects (AMEs), representing the average change in the predicted probability of each outcome category associated with a one-unit change in a given predictor, holding all other variables constant.**

|  | Non-responders | Early attritors | Gradual attritors | Mid-wave attritors | Good responders | Stayers |
| --- | --- | --- | --- | --- | --- | --- |
| ***Big-5 Personality Traits*** |  |  |  |  |  |  |
| Conscientiousness Score | -0.000 | -0.001 | -0.002* | -0.000 | -0.003*** | 0.007*** |
|  | [-0.001,0.000] | [-0.002,0.001] | [-0.004,-0.001] | [-0.002,0.001] | [-0.005,-0.002] | [0.005,0.010] |
| Openness Score | 0.001 | 0.001 | 0.002* | 0.002 | -0.001 | -0.004*** |
|  | [0.000,0.001] | [-0.000,0.002] | [0.001,0.004] | [0.000,0.003] | [-0.003,0.000] | [-0.006,-0.002] |
| Extroversion Score | -0.000 | 0.002*** | 0.001 | 0.001 | -0.000 | -0.004*** |
|  | [-0.001,0.000] | [0.001,0.004] | [-0.001,0.002] | [-0.000,0.002] | [-0.001,0.001] | [-0.006,-0.002] |
| Neuroticism Score | 0.000 | 0.002** | 0.002** | 0.001 | 0.001 | -0.006*** |
|  | [-0.000,0.001] | [0.001,0.004] | [0.001,0.004] | [-0.001,0.002] | [-0.001,0.002] | [-0.008,-0.004] |
| Agreeableness Score | 0.000 | 0.002 | 0.000 | -0.001 | 0.003*** | -0.004*** |
|  | [-0.000,0.001] | [0.000,0.003] | [-0.001,0.002] | [-0.002,0.001] | [0.002,0.005] | [-0.007,-0.002] |
| ***Panel Tenure***  ***(Ref: Less than 1 year)*** |  |  |  |  |  |  |
| 1 year and above | 0.001 | -0.011 | -0.016 | 0.011 | -0.020 | 0.035 |
|  | [-0.005,0.006] | [-0.029,0.007] | [-0.036,0.004] | [-0.008,0.030] | [-0.042,0.002] | [0.004,0.067] |
| ***Hispanic***  ***(Ref: No)*** |  |  |  |  |  |  |
| Yes | 0.008 | 0.002 | 0.008 | -0.017 | 0.042** | -0.044* |
|  | [-0.000,0.017] | [-0.016,0.021] | [-0.013,0.029] | [-0.036,0.003] | [0.017,0.068] | [-0.079,-0.010] |
| ***Race & Ethnicity***  ***(Ref: White only)*** |  |  |  |  |  |  |
| Black only | 0.003 | 0.019 | -0.002 | -0.011 | 0.049** | -0.058** |
|  | [-0.006,0.012] | [-0.005,0.043] | [-0.027,0.022] | [-0.034,0.012] | [0.019,0.079] | [-0.097,-0.019] |
| Others | 0.007 | -0.022 | -0.010 | -0.009 | 0.029 | 0.005 |
|  | [-0.002,0.015] | [-0.040,-0.003] | [-0.032,0.012] | [-0.032,0.014] | [0.003,0.055] | [-0.031,0.042] |
| ***Gender***  ***(Ref: Female)*** |  |  |  |  |  |  |
| Male | 0.003 | 0.017 | 0.006 | -0.009 | 0.008 | -0.025 |
|  | [-0.002,0.008] | [0.002,0.032] | [-0.011,0.022] | [-0.025,0.007] | [-0.010,0.026] | [-0.051,0.001] |
| ***Age Group***  ***(Ref: 18-44)*** |  |  |  |  |  |  |
| 45-64 | -0.006 | -0.072*** | -0.065*** | -0.030** | -0.039*** | 0.212*** |
|  | [-0.011,-0.001] | [-0.089,-0.056] | [-0.084,-0.045] | [-0.049,-0.011] | [-0.060,-0.019] | [0.182,0.243] |
| 65+ | 0.000 | -0.075*** | -0.091*** | -0.046*** | -0.052*** | 0.263*** |
|  | [-0.010,0.011] | [-0.097,-0.054] | [-0.113,-0.069] | [-0.070,-0.022] | [-0.078,-0.025] | [0.223,0.304] |
| ***Education***  ***(Ref: GED or high school)*** |  |  |  |  |  |  |
| Some College | 0.003 | 0.012 | -0.027* | -0.009 | 0.017 | 0.005 |
|  | [-0.003,0.008] | [-0.007,0.031] | [-0.047,-0.007] | [-0.029,0.011] | [-0.005,0.039] | [-0.026,0.037] |
| College and above | 0.003 | -0.022 | -0.022 | -0.015 | -0.016 | 0.071*** |
|  | [-0.003,0.010] | [-0.040,-0.003] | [-0.044,0.000] | [-0.036,0.006] | [-0.038,0.006] | [0.038,0.104] |
| ***HH Income***  ***(Ref: Below $50K)*** |  |  |  |  |  |  |
| $50-$75K | -0.004 | -0.006 | -0.020 | -0.002 | 0.009 | 0.023 |
|  | [-0.011,0.003] | [-0.026,0.014] | [-0.042,0.002] | [-0.023,0.020] | [-0.015,0.033] | [-0.012,0.057] |
| $75K and above | -0.005 | -0.016 | -0.017 | -0.005 | -0.006 | 0.049** |
|  | [-0.011,0.002] | [-0.035,0.002] | [-0.037,0.004] | [-0.026,0.016] | [-0.028,0.016] | [0.016,0.082] |
| ***Employment Status***  ***(Ref: Currently working)*** |  |  |  |  |  |  |
| Currently not working | -0.004 | -0.030*** | -0.017 | -0.013 | -0.007 | 0.072*** |
|  | [-0.010,0.001] | [-0.046,-0.015] | [-0.035,0.001] | [-0.031,0.005] | [-0.026,0.012] | [0.043,0.101] |
| ***Household Size***  ***(Ref: 1)*** |  |  |  |  |  |  |
| 2 | -0.006 | -0.016 | 0.018 | 0.004 | 0.014 | -0.015 |
|  | [-0.013,0.002] | [-0.039,0.007] | [-0.004,0.040] | [-0.018,0.027] | [-0.010,0.038] | [-0.051,0.022] |
| 3 and above | -0.002 | -0.013 | 0.032* | 0.011 | 0.026 | -0.054** |
|  | [-0.010,0.006] | [-0.035,0.010] | [0.010,0.054] | [-0.012,0.034] | [0.002,0.051] | [-0.091,-0.017] |
| ***Health Status*** |  |  |  |  |  |  |
| Self-report of health | -0.000 | -0.010 | -0.001 | 0.008 | 0.006 | -0.002 |
|  | [-0.003,0.003] | [-0.019,-0.002] | [-0.010,0.008] | [-0.001,0.017] | [-0.004,0.015] | [-0.016,0.012] |
| n | 5,550 | | | | | |

95% confidence intervals in brackets; * p < 0.10, ** p < 0.05, *** p < 0.01. All p values were adjusted for multiple hypothesis tests using Holm’s method. Note the 95% CIs were not adjusted for multiple hypothesis tests.
